# New insights into the structure and function of the complex between the *Escherichia coli* Hsp70, DnaK, and its nucleotide-exchange factor, GrpE

**DOI:** 10.1016/j.jbc.2023.105574

**Published:** 2023-12-16

**Authors:** Maria-Agustina Rossi, Alexandra K. Pozhidaeva, Eugenia M. Clerico, Constantine Petridis, Lila M. Gierasch

**Affiliations:** 1 Department of Biochemistry & Molecular Biology, University of Massachusetts Amherst, Amherst, Massachusetts, USA; 2 Department of Chemistry, University of Massachusetts Amherst, Amherst, Massachusetts, USA

**Keywords:** 70 kDa heat shock protein, chaperone, GrpE, DnaK, NMR, nucleotide exchange factor

## Abstract

The 70 kDa heat shock proteins (Hsp70s) play a pivotal role in many cellular functions using allosteric communication between their nucleotide-binding domain (NBD) and substrate-binding domain, mediated by an interdomain linker, to modulate their affinity for protein clients. Critical to modulation of the Hsp70 allosteric cycle, nucleotide-exchange factors (NEFs) act by a conserved mechanism involving binding to the ADP-bound NBD and opening of the nucleotide-binding cleft to accelerate the release of ADP and binding of ATP. The crystal structure of the complex between the NBD of the *Escherichia coli* Hsp70, DnaK, and its NEF, GrpE, was reported previously, but the GrpE in the complex carried a point mutation (G122D). Both the functional impact of this mutation and its location on the NEF led us to revisit the DnaK NBD/GrpE complex structurally using AlphaFold modeling and validation by solution methods that report on protein conformation and mutagenesis. This work resulted in a new model for the DnaK NBD in complex with GrpE in which subdomain IIB of the NBD rotates more than in the crystal structure, resulting in an open conformation of the nucleotide-binding cleft, which now resembles more closely what is seen in other Hsp/NEF complexes. Moreover, the new model is consistent with the increased ADP off-rate accompanying GrpE binding. Excitingly, our findings point to an interdomain allosteric signal in DnaK triggered by GrpE binding.

Heat shock proteins of 70 kDa (Hsp70s) mediate highly diverse cellular functions to maintain protein homeostasis in all organisms (1, 2). Hsp70s facilitate all these processes by undergoing dramatic conformational rearrangements allosterically triggered by ATP binding and hydrolysis such that their affinities for protein clients are switched from high to low. Central players in the modulation of the Hsp70 allosteric cycle are two classes of cochaperones: J-domain proteins, which stimulate the Hsp70 ATPase activity and thus accelerate the switch from the ATP-bound and low substrate affinity state to the ADP-bound high substrate affinity state; and nucleotide-exchange factors (NEFs), which facilitate the exchange of ADP back to ATP (2, 3).

The conserved structure of Hsp70s is made up of two domains connected by a flexible linker (Fig. 1*A*). The 44 kDa nucleotide-binding domain (NBD) belongs to the actin structural class and is comprised of two lobes formed by four subdomains: IA and IB in lobe I and IIA and IIB in lobe II (2, 3). ATP binds deep in the cleft between the lobes and contacts all four subdomains (Fig. 1*A*). The 30 kDa substrate-binding domain (SBD) is composed of a β-sandwich subdomain (βSBD) that contains the canonical substrate-binding pocket, an α-helical lid that covers the substrate-binding site, and a flexible C-terminal tail (Fig. 1*A*).

**Figure 1:**
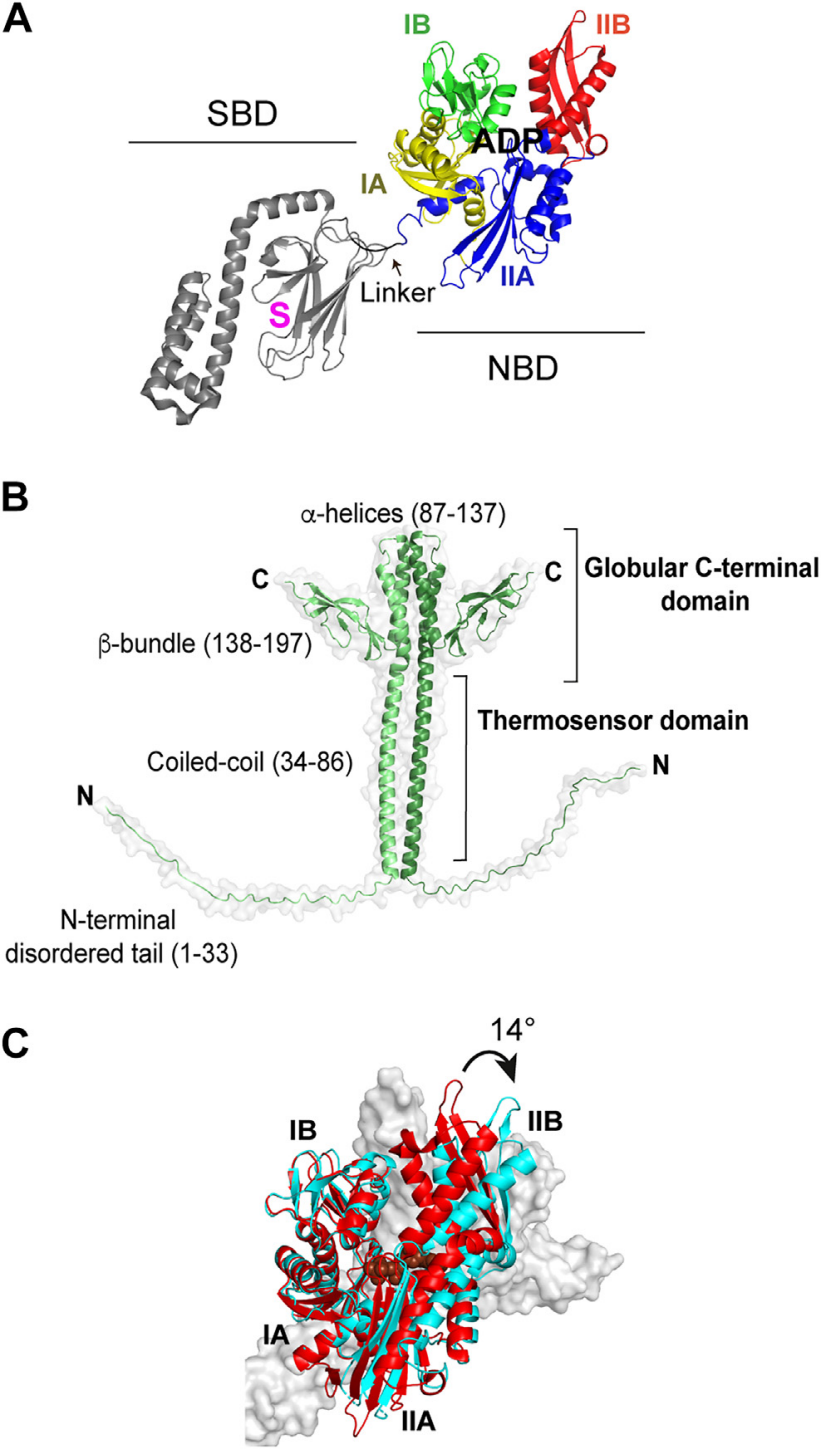
**Allosteric cycle and structural features of DnaK,and structures of thenucleotide-binding domain (NBD) and GrpE.***A*, ADP-bound, domain undocked DnaK (Protein Data Bank [PDB] code: 2KHO) where the structural features are indicated, and the subdomains of the NBD are shown in color (S: substrate-binding site). The flexible C-terminal tail of DnaK is not shown. *B*, AlphaFold-based structure of GrpE where the structural regions are indicated. *C*, overlay of the *Escherichia coli* ATP-bound DnaK NBD (*red*, PDB code: 4B9Q) with the *E. coli* DnaK NBD^1–388^ (*cyan*) bound to GrpE^33–197^ G122D (*gray*, PDB code: 1DKG). Nucleotides are shown in *space fill*.

NEFs for Hsp70s belong to four classes: BAG-1, HspBP1 and Hsp110 in eukaryotes, and GrpE in prokaryotes, mitochondria, and chloroplasts (2, 4). All NEFs accelerate the Hsp70 allosteric cycle by increasing the off-rate of ADP from the NBD (4). Despite their structural and evolutionary diversity, all NEFs utilize the same fundamental mechanism to facilitate nucleotide exchange: They bind to the ADP-bound NBD and open the nucleotide-binding cleft by rotating and shifting of subdomain IIB, facilitating ADP release (3, 4).

The NEF of the bacterial Hsp70 DnaK, GrpE, has a unique structure and function. It adopts a dimeric crucifix structure, consisting of a globular C-terminal domain with two β-bundles and four α-helices, and a long coiled-coil domain (Fig. 1*B*) (5), which unfolds with a melting temperature of 48 °C (6, 7), enabling it to act as a cellular thermosensor (8, 9, 10). Above normal physiological temperatures for *Escherichia coli* (37 °C), GrpE begins to melt and thus loses its NEF function causing DnaK to remain in the ADP-bound high substrate affinity conformation (9).

The crystal structure of a complex between the NBD of the *E. coli* DnaK and GrpE in the absence of nucleotide revealed that GrpE interacts with the NBD *via* its globular C-terminal domain (11). Although dimerization is essential for the functional interaction of GrpE with DnaK (12), only one of the GrpE monomers interacts with the NBD in the structure (11). NBD subdomain IA contacts the upper part of the coiled-coil domain, and subdomains IB and IIB interact with the GrpE β-bundle domain (Fig. 1*C*). In this crystal structure, GrpE binding causes a 14° rotation of subdomain IIB in the NBD (11), whereas in a number of structures of eukaryotic Hsp70/NEF complexes, the rotation of subdomain IIB ranges from 14° to 27° (3, 4). In the DnaK NBD/GrpE crystal structure, GrpE is N-terminally truncated to remove the flexible 33 amino acids and, more importantly, it contains a point mutation, G122D (11), that has been shown to render the NEF inactive (10, 13, 14), raising questions about its use to depict the functional complex. *In vivo*, G122D GrpE inhibits bacterial growth at high temperatures (43.5 °C) and abolishes λ-phage replication at all temperatures (14). *In vitro*, G122D GrpE forms a weak complex with the DnaK NBD, is unable to accelerate nucleotide exchange, and cannot assist DnaK in the refolding of thermally denatured luciferase (10). In addition to concerns about the functional defects associated with the G122D GrpE variant present in the structure of the DnaK NBD/GrpE complex, other observations suggest that this structure may not represent a physiologically active NBD/GrpE complex. For example, mutations in GrpE at sites that contact the NBD in the crystal structure did not impact GrpE function (15), whereas mutations and deletions of DnaK NBD or GrpE at sites away from the binding interface reported in the crystal structure did lead to disruption of the NBD/GrpE complex in solution (10, 16, 17).

In the current study, we have examined the complex between the DnaK NBD and wildtype GrpE using a combination of computational modeling, NMR, mutagenesis, and biochemical assays. The resulting structural model differs from the published crystal structure in ways that make sense, given the position of the GrpE mutation present in the structure, and is consistent with previous mutagenesis and biochemical data (10, 16). In our structural model, the movement of subdomain IIB of the NBD in complex with GrpE is substantially larger than reported in the earlier crystal structure, and this rotation is comparable to that observed for other NBD/NEF complexes (4, 18, 19, 20, 21). Finally, our data suggest that GrpE binding to DnaK influences interdomain communication from the NBD to the SBD.

## Results

### The AlphaFold-predicted structure of the DnaK NBD/GrpE complex differs from the reported crystal structure

As a starting point for our studies of the DnaK/GrpE interaction, we predicted the structure of the complex between DnaK NBD (1–392, NBD^1–392^) and full-length wildtype GrpE using AlphaFold-multimer (22, 23). The resulting structure has a per-residue confidence score (pLDDT) above 80 for the NBD and the GrpE C-terminal globular domain (Fig. S1) and lower confidence score for the coiled-coil domain and disordered tail region of GrpE (Fig. S1), both known to be relatively flexible (24, 25).

**Figure 2:**
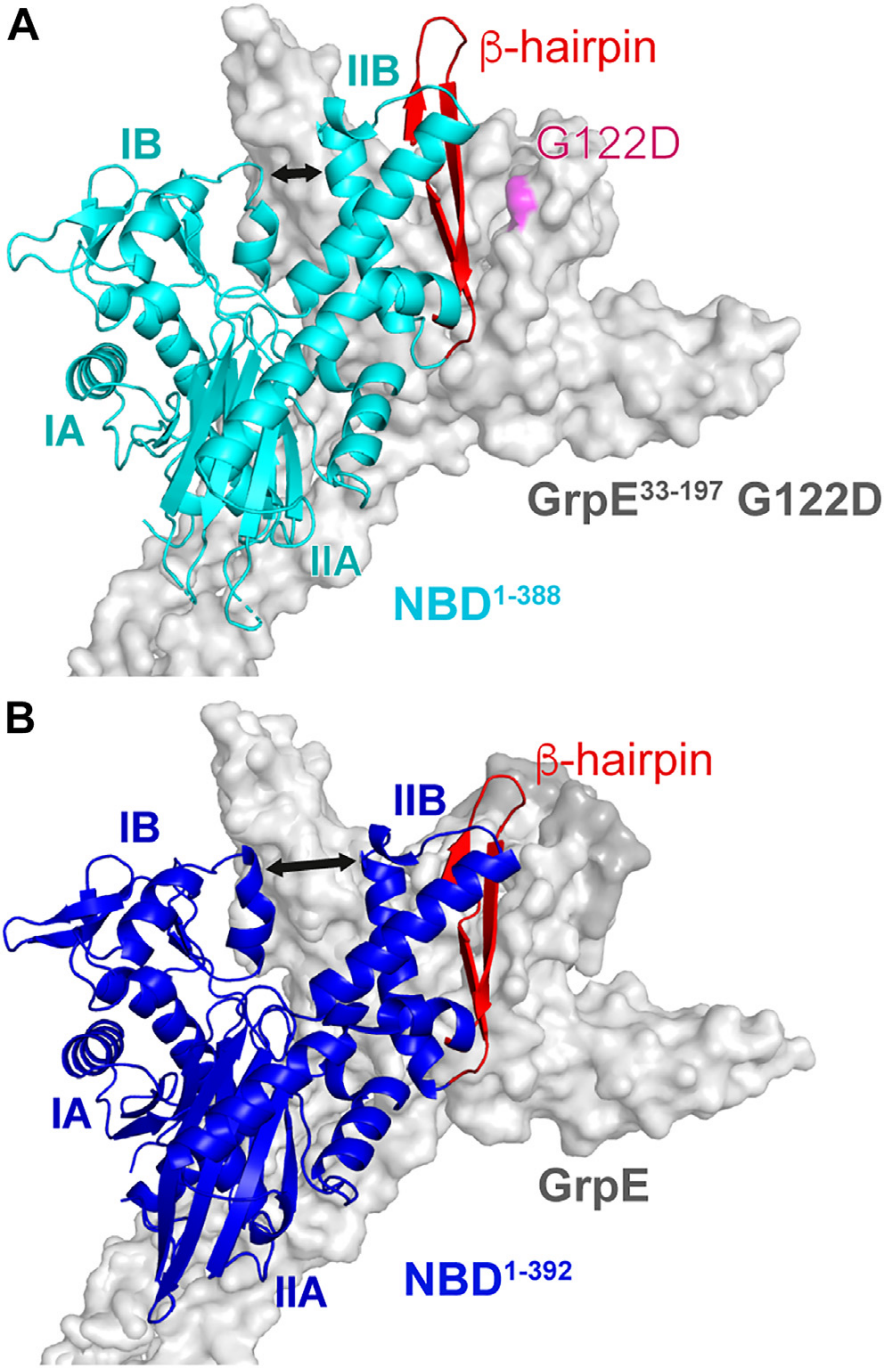
**AlphaFold-predicted model shows a greater rotation of nucleotide-binding domain (NBD) subdomain IIB than in the crystal structure.***A*, crystal structure of NBD^1–388^/GrpE^33–197^ G122D complex (Protein Data Bank code: 1DKG). The *black arrows* mark the gap between subdomains IB and IIB in both panels. *B*, AlphaFold-predicted structure of the DnaK NBD^1–392^/GrpE complex (residues missing in the crystal structure of NBD/GrpE are depicted in *dark gray*).

Overall, the AlphaFold-predicted structure of the DnaK NBD^1–392^/GrpE complex is similar to the published crystal structure of the NBD^1–38^^8^/GrpE^33–197^ G122D complex (11), but there are important differences. While similar interfaces are formed in both structures between subdomains IA (residues 28–33) and IB (residues 51–61) of the NBD and the β-bundle of GrpE, the conformation of NBD subdomain IIB is different (Figs. 2 and S2). In the crystal structure, the interaction between NBD and GrpE is mediated by an interface formed between the NBD subdomain IIB (residues 255–272) and the GrpE β-bundle (Figs. 2*A* and S2*A*). However, in the AlphaFold model, the NBD subdomain IIB is rotated more toward the GrpE α-helices in comparison with ATP-bound DnaK (Protein Data Bank code: 4B9Q) (Figs. 2*B* and S2*B*). Thus, the NBD β-hairpin (residues 276–302) interacts with the four α-helices of GrpE that form the dimerization interface (residues 94–106 and 117–129), where mutation G122D lies in the crystallized complex (Figs. 2 and S2) (6, 26, 27). The greater rotation of subdomain IIB observed in the predicted structure creates a wide gap between the NBD lobes, similar to that seen in the structures of eukaryotic NBD/NEF complexes (4, 18, 19, 20, 21).

Our confidence in the AlphaFold-predicted structure of the NBD/GrpE complex was buoyed by the fact that it offers a compelling explanation for the substantial functional impairment caused by the G122D mutation (10) and for the puzzling absence of functional impact observed upon mutation of the β-hairpin of GrpE (16). Consequently, we endeavored to validate the NBD/GrpE AlphaFold-predicted structure through direct experimental tests.

### NMR chemical shift perturbations between nucleotide-free DnaK NBD^1–392^ and NBD^1–392^ in complex with GrpE are consistent with the AlphaFold-predicted structure

We tested the AlphaFold model of the DnaK NBD^1–392^/GrpE complex using solution NMR chemical shift perturbations (CSPs), which report on local and global structural changes between different states of a protein with high sensitivity (28, 29). CSPs result from either conformational changes or alterations in the chemical environment of a residue. Complex formation between two proteins can cause both phenomena, as local perturbations may arise when interfaces form or when conformational changes occur upon binding. Therefore, CSPs are excellent reporters to characterize the DnaK/GrpE complex in solution. To identify the residues in the DnaK NBD affected by GrpE binding, we exploited two isotope labeling strategies: uniform labeling of NBD^1–392^ with ^15^N or selective labeling of methyl groups of Ile, Leu, and Val (ILV) residues with ^13^C and ^1^H in an otherwise deuterated background (30). ^15^N backbone labeling provides heteronuclear single quantum coherence (HSQC) signals for all residues except proline. However, ^1^H–^15^N spectra are readily broadened by enhanced relaxation because of slow tumbling of species with high molecular weight or asymmetric shape. Both of these issues are relevant for our system given the elongated shape of GrpE (Fig. 1*B*) and the molecular weight of the complex (86 kDa). By contrast, ^13^C-methyl labels of ILV residues provide fewer but sharper signals that are less affected by molecular weight and shape (31). Unless specified, the NBD^1–392^ is nucleotide free, and it carries a mutation (T199A) that minimizes ATP hydrolysis (32).

**Figure 3:**
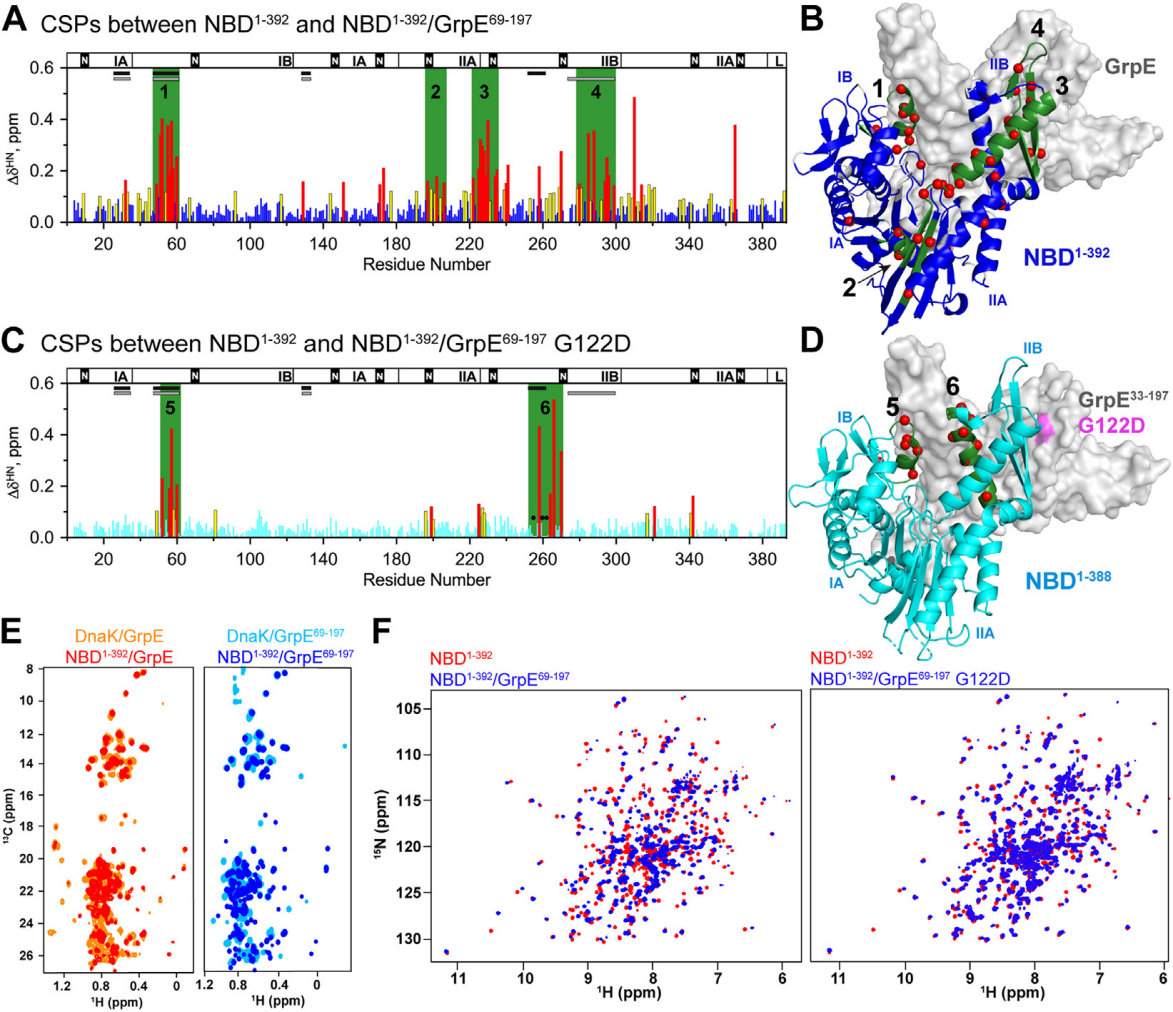
**Chemical shift perturbations (CSPs) between**^**15**^**N NBD**^**1–392**^**and**^**15**^**N NBD**^**1–392**^/**GrpE**^**69–197**^**complex are consistent with the AlphaFold-predicted structure.** Histograms showing CSPs (Δδ^HN^) of the backbone amides between NBD^1–392^ and NBD^1–392^ in complex with GrpE variants (as indicated) as a function of residue number. Data for residues with “large” Δδ^HN^ (>0.15 ppm, which is two times the SD = 0.07) or “significant CSP” (Δδ^H^ or Δδ^N^ larger than 2 SDs) are colored *red* and *yellow*, respectively. Regions of contiguous residues showing large perturbations because of NEF binding are highlighted in *green*; *black circles* mark missing residue resonances in the spectra of the complex. *Bars* at the top of the histograms indicate the NBD subdomains, the ^389^VLLL^392^ linker (L), and the nucleotide-binding site (*black boxes*, N). *Continuous black* or *gray lines* indicate the NBD/GrpE interaction interfaces observed in the crystal structure or AlphaFold-predicted structure, respectively. *A*, histogram of the CSPs (Δδ^HN^) for the backbone amides between NBD^1–392^ and NBD^1–392^/GrpE^69–197^. *B*, CSPs from (*A*) mapped in the AlphaFold-predicted structure of the NBD^1–392^/GrpE complex. *C*, histogram of the CSPs (Δδ^HN^) for the backbone amides between NBD^1–392^ and NBD^1–392^/GrpE^69–197^ G122D. *D*, data from (*C*) depicted in the crystal structure of the NBD^1–388^/GrpE^33–197^ G122D complex (Protein Data Bank code: 1DKG). *E*, GrpE in complex with ILV ^13^C-methyl-labeled NBD^1–392^ or ILV ^13^C-methyl-labeled DnaK (*left*) and GrpE^69–197^ in complex with ILV ^13^C-methyl-labeled NBD^1–392^ or ILV ^13^C-methyl-labeled DnaK (*right*). *F*, ^1^H–^15^N HSQCs of ^15^N-NBD^1–392^ and ^15^N-NBD^1–392^/GrpE^69–197^ complex (*left*) and ^15^N-NBD^1–392^ and ^15^N-NBD^1–392^/GrpE^69–197^ G122D complex (*right*). For the chemical shift data, see Tables S1–S3. HSQC, heteronuclear single quantum coherence; ILV, Ile, Leu, and Val; NBD, nucleotide-binding domain; NEF, nucleotide-exchange factor.

To reduce the molecular weight of the complex and make its shape more globular, thus improving the spectral properties of the complex, we created a truncated GrpE variant, GrpE^69–197^, which lacks the N-terminal disordered tail and a portion of the coiled-coil domain (Fig. S3). The heteronuclear multiple quantum coherence spectra of ILV ^13^C-methyl-labeled NBD^1–392^ or full-length DnaK in complex with GrpE or GrpE^69–197^ overlay closely, validating the use of the truncated construct (Figs. 3 and S3, chemical shift data are provided in Tables S1 and S2).

The CSPs of ^15^N NBD^1–392^ observed upon formation of the NBD^1–392^/GrpE^69–197^ complex mapped to contiguous structural regions (Fig. 3, *A* and *B*). Residues 49 to 60 in subdomain IA and residues 31 to 33 in subdomain IB of the NBD show perturbations that are expected based on both the crystal and the AlphaFold-predicted structures, as they are located on the NBD/GrpE interfaces formed in both structures (Fig. S2). In the crystallized complex, residues 255 to 272 of the α-helix in subdomain IIB are in close contact with the β-bundle of GrpE, and the loop formed by residues 131 to 134 interacts with the GrpE coiled-coil domain (Fig. S2*A*), yet no CSPs were observed in these regions (Fig. 3, *A* and *B*). In contrast, we observed CSPs corresponding to the NBD β-hairpin (residues 276–302, Fig. 3, *A* and *B*) that is in intimate contact with GrpE in the AlphaFold-predicted structure but not in the crystal structure (Figs. 2, and S2). The G122D mutation in GrpE present in the crystallized complex lies on the face of GrpE that forms the contact with the NBD 276 to 302 β-hairpin in the predicted structure, providing a potential explanation for the difference between the crystal and the AlphaFold-predicted structures (Fig. 2).

To test whether the GrpE G122D mutation led to the differences between the AlphaFold-predicted structure (supported by NMR data) and the crystal structure of DnaK NBD^1–388^/GrpE^33–197^ G122D, we obtained the ^1^H-^15^N HSQC spectrum of the ^15^N NBD^1–392^/GrpE^69–197^ G122D complex (Fig. 3). CSPs between NBD^1–392^ and NBD^1–392^/GrpE^69–197^ G122D were only observed in residues 49 to 60 in subdomain IB and in residues 255 to 272 on the α-helix of subdomain IIB (Fig. 3, *C* and *D*). These residues form contacts with GrpE both in the AlphaFold-based structure and in the structure of the crystallized complex (Fig. S2). By contrast, no CSPs were observed in the β-hairpin of subdomain IIB (Fig. 3, *C* and *D*), which forms contacts with GrpE in the AlphaFold-predicted structure and showed CSPs in the NBD/GrpE complex but does not contact GrpE in the crystallized complex (Figs. 2 and S2). Thus, the CSPs observed in the context of the NBD complex with GrpE harboring the G122D mutation are entirely consistent with the interfaces reported in the crystal structure (Fig. S2). Together with the previous reports showing that the G122D mutation abolishes GrpE activity (10), these results suggest that the crystal structure of NBD^1–388^/GrpE^33–197^ G122D does not depict a functional complex. Our data argue that the AlphaFold-predicted structure, supported by our NMR data on the wildtype GrpE^69^^–197^/NBD^1–392^ complex, depicts the functional interaction between the DnaK NBD and its NEF in solution.

### Impacts of mutagenesis support the AlphaFold- and NMR-based model of the NBD/GrpE complex

A key difference between the crystal structure of NBD^1–388^/GrpE^33–197^ G122D and the complex with wildtype GrpE predicted by AlphaFold and supported by NMR CSPs is the position of the 276 to 302 β-hairpin in subdomain IIB of the NBD. Because this hairpin interacts with the α-helical domain of GrpE in the AlphaFold-based structure but not in the crystal structure (Figs. 2 and S2), we reasoned that mutations in this loop would affect NBD/GrpE complex formation if the complex assumes the AlphaFold-predicted structure. Two NBD variants were designed to test this prediction (Fig. S4): NBD^1–392^ Y285R, as Y285 is positioned between GrpE α-helices and surrounded by hydrophobic residues in the predicted complex, and NBD^1–392^ Δ285 to 295, where most of the β-hairpin is deleted. The *K*_*D*_, *k*_on_, and *k*_off_ of the complexes between these NBD^1–392^ variants and GrpE were determined using surface plasmon resonance (SPR) (Table 1 and Fig. S5). Both Y285R and Δ285 to 295 NBD mutations weakened the stability of the complex with GrpE as indicated by their higher *K*_*D*_s (largely attributable to lower *k*_on_ rates compared with the wildtype NBD/GrpE complex). These results further support the validity of the AlphaFold-based structure of the NBD/GrpE complex.

**Table 1:** Impact of residue substitutions in subdomain IIB of the NBD on the NBD^1–392^/GrpE complex

Complex	*K*_*D*_ (nM)	*k*_on_ (s^−1^ M^−1^)	*k*_off_ (s^−1^)	Expected effect	
				Crystal^a^	Predicted^b^
NBD^1–392^/GrpE	3.8 ± 0.6	(5.9 ± 0.3) × 10^6^	(2.1 ± 0.2) × 10^−2^	—	—
NBD^1–392^Q260R/GrpE	4.1 ± 0.3	(5.6 ± 0.2) × 10^6^	(2.31 ± 0.05) × 10^−2^	Yes	—
NBD^1–392^Y285R/GrpE	120 ± 10	(2.4 ± 0.1) × 10^4^	(1.78 ± 0.02) × 10^−2^	—	Yes
NBD^1–392^Δ285–295/GrpE	440 ± 60	(3.9 ± 0.3) × 10^4^	(1.58 ± 0.06) × 10^−2^	—	Yes

*K*_*D*_, *k*_on_, and *k*_off_ determined by SPR^c^.

a Crystal structure of NBD^1–388^/GrpE^33–197^ G122D (PDB code: 1DKG).

b AlphaFold-predicted structure of NBD^1–392^/GrpE.

c Sensorgrams are shown in Fig. S5.

Concurrently, we also tested NBD mutation Q260R. Residues 255 to 266 of the α-helix of subdomain IIB interact with the β-bundle of GrpE in the crystal structure of the complex but not in the AlphaFold-predicted structure. Therefore, we expected that a Q260R mutation in the NBD would disrupt formation of the NBD/GrpE complex only if its conformation closely resembled that in the crystal structure (Fig. S4). We found that formation of the NBD^1–392^ Q260R/GrpE complex was characterized by *K*_*D*_, *k*_on_, and *k*_off_ parameters identical within error to those for the wildtype NBD/GrpE complex (Table 1). We conclude that the α-helix of subdomain IIB is not part of the interaction interface with GrpE.

### GrpE binding to DnaK affects interdomain allostery

Our NMR analysis of CSPs in the DnaK NBD upon formation of the GrpE complex suggests that GrpE binding may cause both local (intradomain) and long-range (interdomain) conformational changes. We observed CSPs in NBD^1–392^/GrpE^69–197^ that mapped in regions other than the interaction interfaces seen in the AlphaFold-predicted structure of the complex (Fig. 3*A*). In subdomain IIB, α-helix 228 to 243 shows many CSPs, and in subdomain IIA, CSPs are observed in the small loop that connects α-helix 228 to 243 with the β-sheets, and near the crossing helices that form a pocket for interdomain linker binding (33, 34, 35, 36, 37). In addition, the subdomain interfaces between IIB and IIA, and between subdomain IIA and the α-helix 171 to 182 in IA, also exhibit CSPs. All these intradomain perturbations are indicative of subdomain rearrangements previously described to be important for allosteric signal transduction within the NBD (34). Particularly exciting is the path of CSPs connecting the β-hairpin of subdomain IIB with the crossing helices and the hydrophobic pocket where the interdomain linker docks, coupling the nucleotide-dependent allosteric conformational change to the SBD and essential for Hsp70 function (37).

**Figure 4:**
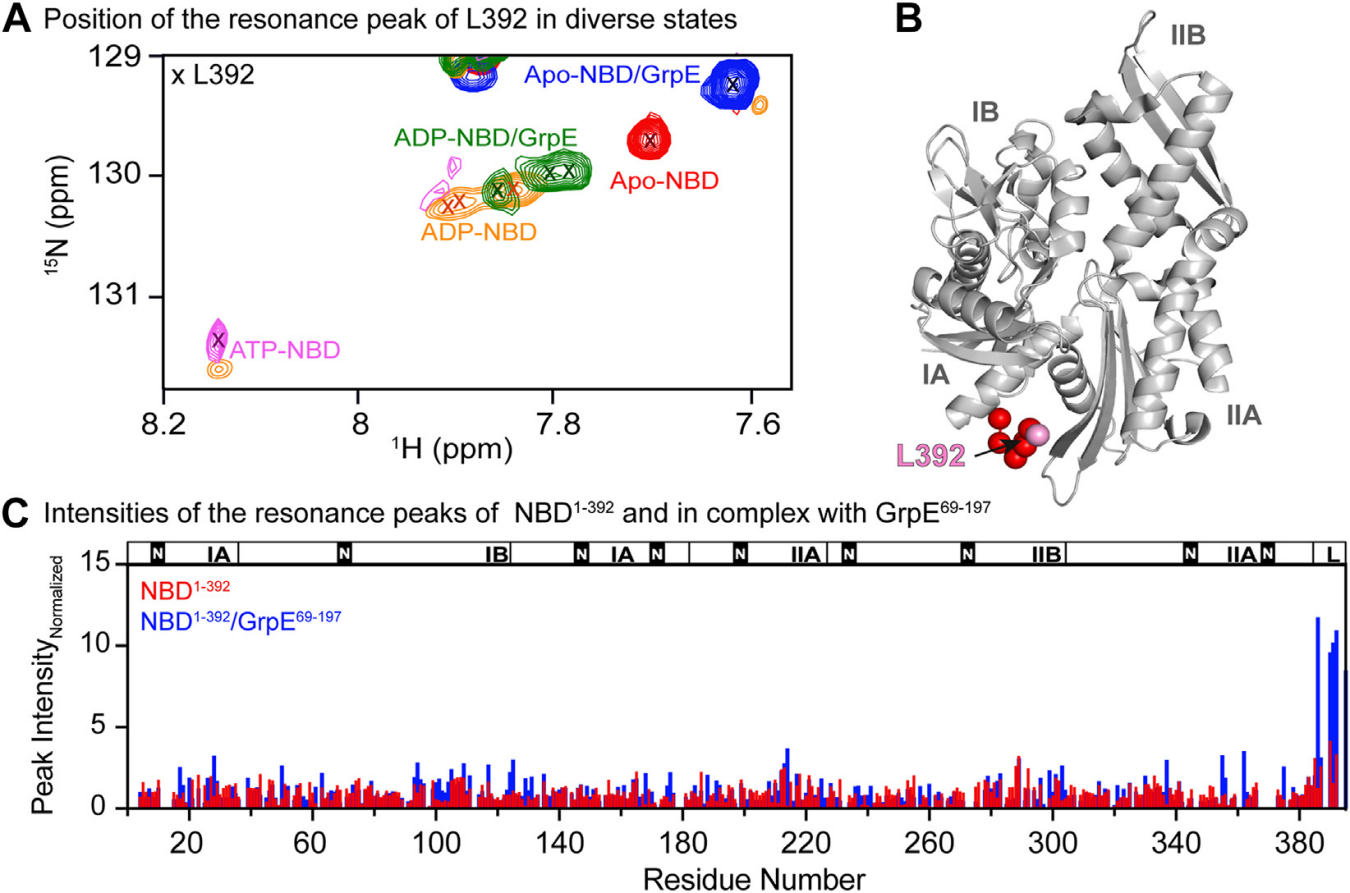
**Docking of the DnaK interdomain linker on the nucleotide-binding domain (NBD) followed by NMR.***A*, chemical shift of ^1^H–^15^N L392 at diverse ligand-bound states of the NBD^1–392^. (For simplicity, we refer to “NBD” instead of NBD^1–392^.) The nucleotide state of NBD^1–392^ for each resonance is indicated. *B*, structure of ATP-bound NBD^1–392^ where the interdomain linker (in *spheres*) is docked in the binding pocket (Protein Data Bank code: 4B9Q). *C*, normalized intensities of the ^1^H–^15^N resonance peaks of nucleotide-free NBD^1–392^ (*red*) and in complex with GrpE^69–197^ (*blue*). Peak intensities are obtained from the peak height. Because of the differences in the molecular weight of the NBD *versus* NBD/GrpE complex, the peak intensities were normalized based on the mean of each dataset (the mean was calculated from all resonances in the protein, excluding the 10% of the highest and lowest values).

The conformational changes associated with the intradomain allosteric mechanism of the NBD were previously described in a study comparing various nucleotide-bound states of the NBD (34). This study showed that the conformational changes of subdomain IIB are communicated to the interdomain linker binding pocket, and that the chemical shifts and line width of the signals corresponding to the linker residues are exquisitely sensitive to the extent of the linker binding to the hydrophobic pocket on the NBD (34). Based on this correlation, we examined the ^1^H^15^N NMR resonance of interdomain linker residue L392 in unbound NBD^1–392^ and in the NBD^1–392^/GrpE^69–197^ complex (Fig. 4*A*). As seen previously (34, 35, 36, 37), ATP binding to the NBD causes linker docking into the pocket under the crossing helices, broadening the L392 resonance and shifting it significantly to lower field compared with its resonance position in the apo-NBD. The interpretation of these shifts was that the linker was unbound in apo-NBD and bound to the hydrophobic pocket in ATP-bound NBD (34). Strikingly, in the NBD^1–392^/GrpE^69–197^ complex, the resonance for L392 is shifted even further upfield than in apo-NBD (Fig. 4*A*). Furthermore, the resonances corresponding to the hydrophobic residues of the interdomain linker in the NBD^1–392^/GrpE^69–197^ complex were narrower and had higher intensity than in apo-NBD^1–392^, consistent with increased dynamics (Fig. 4, *B* and *C*). Together, these results suggest that GrpE binding causes an intradomain conformational change propagating from subdomain IIB to the crossing helices, closing the binding pocket and preventing linker docking even more than in any other conformational state of NBD previously characterized.

**Figure 5:**
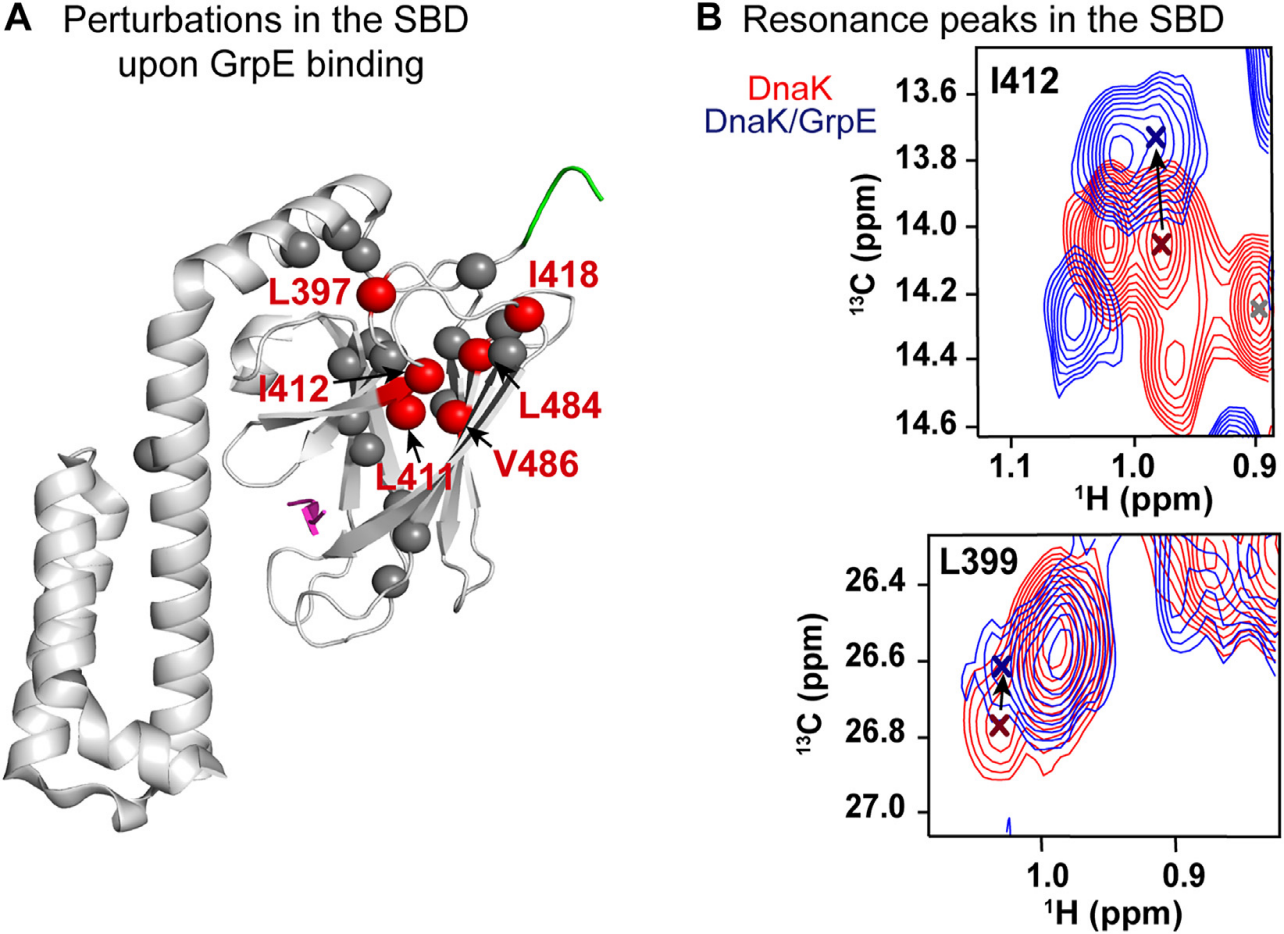
**Perturbations are observed in the SBD upon GrpE binding to full-length DnaK.***A*, representative ILV ^13^C-methyl-labeled residues in the SBD that showed perturbations upon GrpE binding to full-length DnaK are shown as *red spheres*, and *gray spheres* represent unperturbed residues. The substrate is shown in *pink* and interdomain linker in *green* (Protein Data Bank code: 7N6M). *B*, zoomed-in regions of the spectra in Fig. S8 where resonances assigned to the SBD shift upon GrpE binding (residues L399 and I412). ILV, Ile, Leu, and Val; SBD, substrate-binding domain.

Allostery in DnaK relies on transmission of intradomain ligand–mediated conformational shifts to interdomain signals and consequent conformational remodeling. The observation that GrpE binding strongly disfavors linker docking raises the possibility that the NEF binding signal is communicated to the SBD. We measured CSPs between ILV ^13^C-methyl labeled full-length DnaK and its complex with GrpE (Figs. 5*A* and S6) and found that indeed multiple small CSPs in the SBD were present in the complex compared with free full-length DnaK (Fig. 5*B*). Work in progress will elucidate in greater detail the nature of the signal transmitted to the SBD upon NEF binding.

### The ADP-bound NBD/GrpE complex is of low stability, likely resembles the crystallized apo-complex, and samples multiple conformations en route to the stable nucleotide-free complex

**Figure 6:**
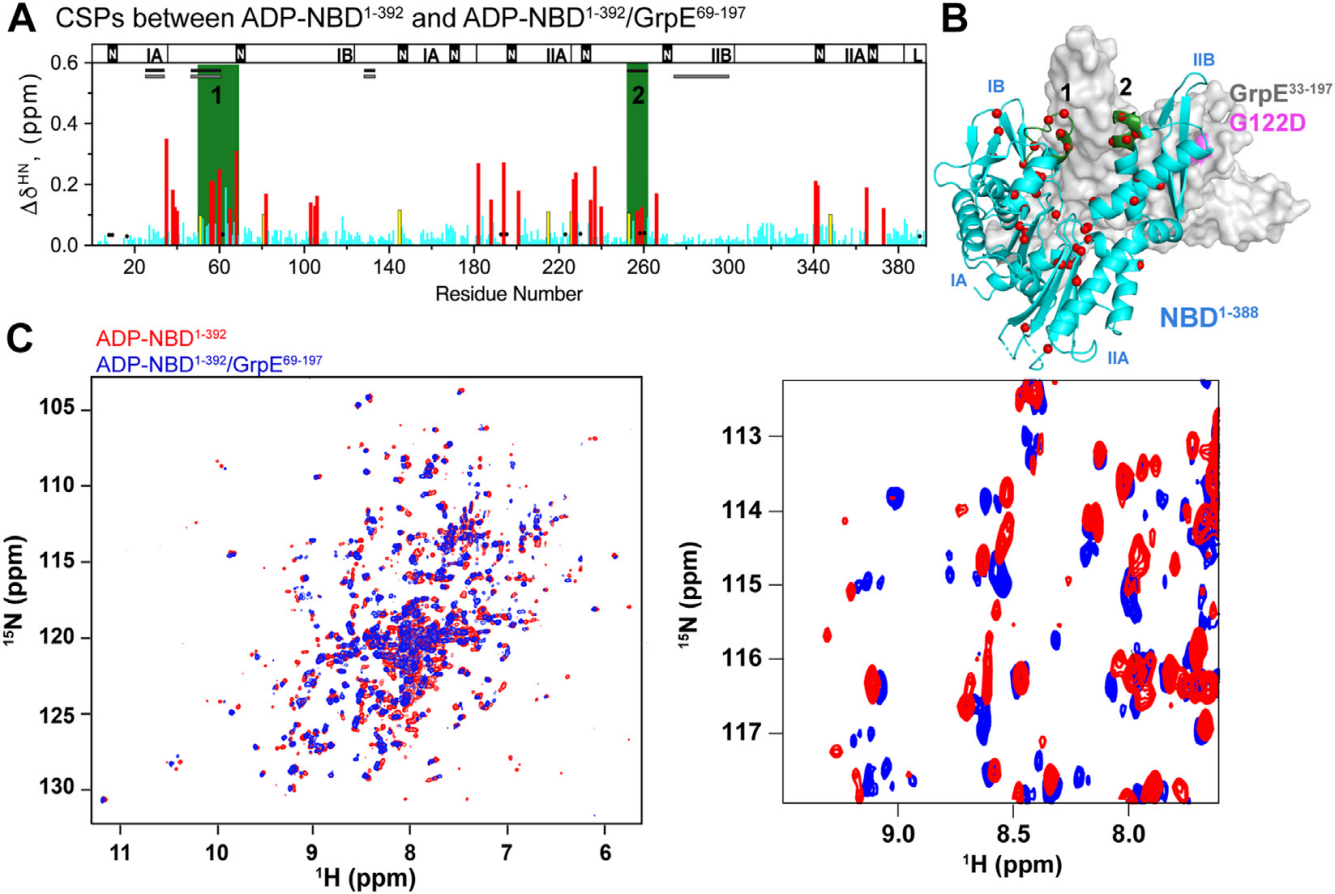
**Chemical shift perturbations (CSPs) between ADP-bound**^**15**^**N NBD**^**1–392**^**and ADP-bound**^**15**^**N NBD**^**1–392**^**in complex with GrpE**^**69–197**^**.***A*, histogram of the CSPs (Δδ^HN^) for the backbone amides between ADP-bound ^15^N NBD^1–392^ alone and in complex with GrpE^69–197^ as a function of residue number [residues with large Δδ^HN^ (>0.15 ppm) or significant CSP (with Δδ^H^ or Δδ^N^ value larger than two SDs) are colored *red* and *yellow*, respectively]; the *green rectangles* highlight regions that are most affected by NEF binding, *black spheres* denote the residues with missing resonances in the spectrum of the complex; *continuous black or gray lines* indicate the NBD/GrpE interaction interfaces observed in the crystal structure or AlphaFold-predicted structure, respectively; the *top bar* shows NBD subdomains, the ^389^VLLL^392^ linker motif (L), and the nucleotide-binding site (*black*, N). *B*, residues with large Δδ^HN^ and residues with missing resonances in the complex are mapped on the crystal structure of NBD^1–388^/GrpE^33–197^ G122D (Protein Data Bank code: 1DKG) as red and *gray spheres*, respectively. *C*, ^1^H–^15^N HSQCs of ADP-bound ^15^N NBD^1–392^ and ADP-bound ^15^N NBD^1–392^/GrpE^69–197^ complex (*left*) and a zoomed-in region of the spectra (*right*). The zoomed-in region is reproduced in Fig. S7, where the label of each resonance is included. For the chemical shift data, see Table S4. HSQC, heteronuclear single quantum coherence; NEF, nucleotide-exchange factor.

Binding of the DnaK NBD to GrpE was reported to proceed *via* a two-step mechanism (38): In the first step, GrpE binds weakly to ADP-bound DnaK forming the ADP-bound DnaK/GrpE complex, and a subsequent conformational change in the NBD leads to the release of ADP and formation of a more stable complex between apo-DnaK and GrpE. To see whether we could observe the initial weak complex that is predicted to occur in this mechanism, we recorded the ^1^H-^15^N HSQC spectrum of ADP-bound ^15^N NBD^1–392^/GrpE^69–197^ (Fig. 6, chemical shift data provided in Table S4). To populate the weak complex enough to observe its ^1^H-^15^N HSQC signals, it was necessary to add a stoichiometric excess of GrpE^69–197^ and Mg-ADP over NBD^1–392^, consistent with the expected instability of this complex relative to the nucleotide-free NBD^1–392^/GrpE^69–197^ complex. Indeed, previous work reported a *K*_*D*_ of 220 nM for the ternary complex (38, 39)—substantially higher than the *K*_*D*_ of the nucleotide-free complex (3.8 ± 0.6 nM, Table 1).

The ^1^H-^15^N HSQC of ADP-bound ^15^N NBD^1–392^/GrpE^69–197^ shows peak duplication and line broadening (Figs. 6, and S7), usually associated with exchange among multiple conformations in the microsecond–millisecond time scale (40). Many CSPs of ADP-bound NBD^1–392^ resonances map at continuous structural regions upon formation of the ADP-bound NBD^1–392^/GrpE^69–197^ complex (Fig. 6). Residues 52 to 63 in subdomain IA of the NBD show perturbations consistent with the AlphaFold-based and crystal structures. However, the perturbations of residues 255 to 272 in subdomain IIB are only consistent with the crystallized complex, where the α-helix interacts with the β-bundle of GrpE. Our results suggest that the AlphaFold-predicted structure of NBD^1–392^/GrpE describes the structure of the complex in the absence of nucleotide, whereas the crystal structure of the NBD^1–388^/GrpE^33–197^ G122D represents the conformation that NBD adopts in the presence of excess Mg-ADP and GrpE.

## Discussion

The allosteric landscape of Hsp70s is formed by linking two domains that each undergo ligand-induced conformational changes. These conformational changes enable the domains to communicate *via* an interdomain linker and interaction interfaces. Moreover, the allosteric landscapes of the individual domains are modulated by cochaperones (2). In particular, the critical conformational switching within the NBD, which is tuned by nucleotide binding, is influenced by interaction with NEFs. Upon binding of ADP or ATP, the cleft between the two lobes of the actin-like NBD fold closes, with subdomain IIB contacting subdomain IB. NEF binding causes a rotation of NBD subdomain IIB, which opens the nucleotide-binding cleft, increasing the nucleotide off-rates. Despite their structural divergence, all NEFs exploit the same mechanism to accelerate ADP to ATP exchange, which otherwise slowly progress through the Hsp70 allosteric cycle (2, 4, 41). The crystal structure of the *E. coli* DnaK NBD/GrpE complex (11) has played a pivotal role in shaping our understanding of NEF action in this system. However, GrpE in the crystallized complex contains a mutation (G122D), reported to render the NEF inactive (10, 13, 14). In this structure, subdomain IIB of the NBD shows only a modest opening of nucleotide-binding cleft compared with many structures of other Hsp70/NEF complexes (4, 19) (Fig. 1). Here, we obtained an AlphaFold-predicted structure of the DnaK NBD/GrpE complex and fully validated it by solution NMR spectroscopy and mutagenesis. The resulting structure differs from the published crystal structure in the extent of rotation of subdomain IIB. In solution and in the AlphaFold structure, the fully open conformation of the nucleotide-binding cleft is achieved by the interaction between β-hairpin 275 to 302 of the NBD and the four α-helices of GrpE, at the interface of the dimerization domain (Fig. S2). This interaction explains why GrpE is only active as a dimer (26, 27), which cannot be deduced from the crystal structure, since in the structure the NBD interacts with only one of the GrpE monomers. In agreement with these observations, DnaK subfamily members that have GrpE as their specific NEF conserve a long β-hairpin, whereas Hsc70 and HscA subfamily members share a 4- or 10-residue shorter β-hairpin (16) supporting the idea that interaction of the β-hairpin 275 to 302 with GrpE is pivotal for NEF function. Moreover, the AlphaFold-predicted structure supported by our experimental data provides an explanation for previous results on the functional impact of NBD or GrpE mutations (10, 16). In addition, the new structure is close to that of a related DnaK with its homolog of GrpE, the *Geobacillus kaustophilus* DnaK/GrpE crystallized complex (Fig. S8) (24).

Excitingly, our study of the DnaK/GrpE complex offers a structural explanation for the proposed mechanism for GrpE-mediated nucleotide exchange of DnaK (38). In this mechanism, GrpE binds to ADP-bound DnaK and stimulates nucleotide dissociation up to 5000-fold (16, 38) *via* a two-step mechanism (38), first forming a weak ternary complex (ADP-bound DnaK/GrpE) where DnaK undergoes a fast conformational change (127 s^−1^, an 8 ms/event) (ADP-bound DnaK^∗^/GrpE). This complex releases ADP, forming the more stable GrpE complex with nucleotide-free DnaK (DnaK/GrpE) (38). We observed an ^1^H-^15^N HSQC spectrum for the ADP-bound ^15^N NBD^1–392^/GrpE^69–197^ complex with considerable line broadening and loss of resonances, indicative of dynamics in the microsecond–millisecond time scale and the presence of multiple conformations. Interestingly, CSPs for the main conformation populated by ADP-bound NBD^1–392^/GrpE^69–197^ were consistent with the crystallized structure, suggesting that the crystal structure represents the initial complex formed between GrpE and ADP-bound DnaK described by Packschies *et al.* (38). After the NBD conformational change induced by GrpE binding and immediately before ADP release, the ADP-bound DnaK^∗^/GrpE complex has a *K*_*D*_ of 11 nM (38), comparable to the *K*_*D*_ we measured for nucleotide-free NBD^1–392^/GrpE (3.8 nM, Table 1) and DnaK/GrpE (3 nM, data not shown), consistent with the presence of similar interaction interfaces in all complexes. Finally, ATP binding disrupts the DnaK/GrpE complex and closes the nucleotide-binding cleft of the DnaK NBD.

Previous studies from our laboratory demonstrated that the allosteric signal triggered by nucleotide binding to the NBD propagates from subdomain IIB through the domain to gate the pocket that serves as docking site for the interdomain linker (34) and then to the SBD (42). In particular, we observed by NMR that in the absence of nucleotide the interdomain linker is mobile, thus undocked from the NBD (Fig. 4, *A*–*C*) (34). We found that ATP binding led to marked reduction in mobility of the linker as it bound into the hydrophobic pocket. Based on the substantial change in line width and chemical shifts of linker residues, we concluded that these were the extremes of an allosteric interconversion (34). It was thus surprising to find that NEF binding led to even narrower linewidths and larger chemical shifts in the linker resonances than those seen for apo-NBD. We attribute this to a greater rotation of subdomain IIB induced by GrpE binding, which *via* the intradomain allosteric signal transmission network within the NBD favors a conformation where the interdomain linker is further undocked from the NBD. We propose that this observation reflects a gating of the hydrophobic pocket to fully exclude interdomain linker docking.

This allosteric signal is further transmitted to DnaK SBD as shown in Figure 5. Previous studies have pointed to an effect of GrpE binding on substrate release from DnaK SBD (11, 24, 25, 39, 43, 44). In the postulated mechanism, GrpE first interacts with the DnaK NBD *via* its globular C-terminal domain. Then, the coiled-coil domain of GrpE is suggested to interact with DnaK, positioning the N-terminal disordered tails of GrpE to contact the SBD and act as a pseudosubstrate (25, 39). Communication between DnaK NBD and SBD triggered by GrpE binding might coordinate the dissociation of nucleotide and release of the substrate in the context of the allosteric cycle. Based on these previous observations (11, 24, 25, 39, 43, 44) and our data showing CSPs in the DnaK SBD upon GrpE binding (Figs. 5 and S6), we conclude that the binding of the NEF to the NBD mediates an allosteric signal that is transmitted from the NBD to the SBD. Further experiments will allow us to delve more deeply into this fascinating system of interdomain/cochaperone allostery.

## Experimental procedures

Detailed materials and methods are included in the supporting information (extended Experimental procedures section).

### Computational methods

A structural model of the NBD^1–392^/GrpE complex was obtained using AlphaFold 2.2.0 multimer (22), accessed *via* the NMRbox platform (45) using default parameters. The prediction was obtained (1) with full database and (2) without it; both yielded the same model that it is reported here. The predicted model using a full database is shown in the figures presented here. The sequences for *E. coli* DnaK and GrpE were obtained from the UniProt database (46). The highest confidence models were identified in each case from the confidence score (47).

### Protein expression and purification

Wildtype *E. coli* full-length DnaK and NBD^1–392^ T199A were expressed and purified as described previously (37, 48) with minor modifications, listed in supporting information. The NBD^1–392^ construct consists of the DnaK NBD and the interdomain linker; to suppress the ATP hydrolysis, a T199A mutation was included (32). His-tagged GrpE, GrpE^69–197^, and GrpE^69–197^ G122D were expressed in BL21(*DE3*) at 37 °C and purified by nickel–nitrilotriacetic acid (NTA) affinity chromatography. The His tag was cleaved with tobacco etch virus protease, and then the His tag, remaining fused protein, and His-tagged tobacco etch virus protease were separated from GrpE, or variants, with a second Ni–NTA chromatography. GrpE concentrations throughout the article always refer to the dimer.

To prepare ^2^H–^15^N-labeled NBD^1–392^ T199A or ^2^H-labeled GrpE^69–197^, established protocols were followed (34, 42, 49, 50); protein expression was induced for 16 h at 20 °C.

### NMR spectroscopy

All spectra were obtained at 25 °C on a 600 MHz-Bruker Advance spectrometer using a triple-resonance inverse cryoprobe.

For ^1^H-^15^N HSQC experiments, the following constructs were employed: ^2^H–^15^N-labeled NBD^1–392^ T199A and ^2^H-labeled GrpE^69–197^, ^2^H-labeled GrpE^69–197^ G122D, or full-length GrpE. Nucleotide-free spectra were obtained with 300 μM of NBD^1–392^ T199A or NBD^1–392^ T199A–GrpE^69–197^ complex, except in the background of the G122D mutation when 450 μM of GrpE^69–197^ was used. The ^1^H-^15^N HSQCs in the presence of ADP were measured with 300 μM of ADP-bound NBD^1–392^ T199A plus 3 mM of Mg-ADP and 600 μM of GrpE^69–197^.

The heteronuclear multiple quantum coherence spectra were measured with 40 μM ^2^H–^13^C methyl labeled ILV nucleotide-free NBD^1–392^ T199A plus 40 μM GrpE, or its truncated variants or 60 μM ^2^H–^13^C ILV nucleotide-free DnaK plus 80 μM GrpE, or the truncated variants. In the presence of ADP, 10-fold (400 μM) and 2-fold (80 μM) excess of Mg-ADP and GrpE or its variants was used, respectively.

### Chemical-shift analysis

The resonance assignments for NBD^1–392^ or ADP-bound NBD^1–392^ were transferred to the respective GrpE complexes, and CSPs of the backbone ^1^H–^15^N atoms (Δδ^HN^) were calculated using the equation:

$$\Delta \delta^{ΗΝ}=\sqrt{{\left(\Delta \delta_{Η}\right)}^{2}+{\left(0.145\;\Delta \delta_{Ν}\right)}^{2}}$$

where Δδ are the differences in the chemical shifts of a nucleus *i* and 0.145 is the weighting factor for ^15^N based on ratio of the average SD of ^1^H and ^15^N (0.93 and 6.4, respectively) for all common amino acids, except prolines, using the Biological Magnetic Resonance Bank database (51).

### SPR measurements

SPR measurements were done in a Biacore T-200 instrument with NTA sensor chips (Series S sensor chip NTA; Cytiva, catalog no.: BR100532). First the chip was activated with Ni^2+^, and then N-terminal His-tagged GrpE was immobilized. Finally, nucleotide-free NBD^1–392^ T199A, or its variants, were perfused at diverse concentrations depending on the *K*_*D*_. Finally, the sensor chip was regenerated with a metal-chelating agent and extensively washed with the running buffer. The control sensorgram consisted in the same steps and settings, but instead of GrpE, running buffer was perfused. Each binding data point represents the experimental sensorgram minus the control.

Each dataset was fit with two different methods: (1) the response units (RUs) at binding equilibrium (RU_eq_) were measured at each NBD (or its variants) concentrations and were fitted with the hyperbola function RU_eq_ = (RU_max_∗[NBD])/(*K*_*D*_ + [NBD]); (2) the association and dissociation of each sensorgram were fitted according with the equations,

$$Association\;{\text{RU}}_{a}=\frac{{\text{RU}}_{\max}\times \left[\text{NBD}\right]}{K_{D}+\left[\text{NBD}\right]}\left(1-e^{-k_{\text{obs}}\times t}\right),k_{\text{obs}}=\left[\text{NBD}\right]{\;k}_{\text{on}\;}{+k}_{\text{off}}$$

$$Dissociation\;{\text{RU}}_{d}={\text{RU}}_{0}\;e^{-\;k_{\text{off}}\times \left(t-t_{0}\right)\;}$$

## Data availability

All data are included in this article or the supporting information, except for the structure coordinates predicted by AlphaFold, which have been deposited in the ModelArchive database and are retrievable at https://www.modelarchive.org/doi/10.5452/ma-ip14z

## Supporting information

This article contains supporting information (17, 29, 37, 39, 48, 49, 52, 53).

## Conflict of interest

The authors declare that they have no conflicts of interest with the contents of this article.
