# Supplementary material for: New insights into the structure and function of the complex between the Escherichia coli Hsp70, DnaK, and its nucleotide-exchange factor, GrpE
Source: J Biol Chem. 2023 Dec 16;300(1):105574. doi: 10.1016/j.jbc.2023.105574 (PMC10825016; doi:10.1016/j.jbc.2023.105574)
Supplement: Supporting Information [file mmc1.pdf]

Supporting information

New insights into the structure and function of the complex between the *E. coli* Hsp70, DnaK, and its nucleotide-exchange factor, GrpE

Maria-Agustina Rossi[a], Alexandra K. Pozhidaeva[a], Eugenia M. Clerico[a], Constantine Petridis[a], and Lila M. Gierasch[a,b]

Affiliations: [a]Department of Biochemistry & Molecular Biology and [b]Department of Chemistry, University of Massachusetts Amherst, Amherst, MA, 01003, USA.

Running title: Structural model of the GrpE/DnaK

Key words: 70-kilodalton heat shock protein (Hsp70), chaperone, GrpE, DnaK, nuclear magnetic resonance (NMR), nucleotide exchange factor.

For correspondence: Lila M. Gierasch, gierasch@biochem.umass.edu

Present addresses: Alexandra K. Pozhidaeva**:** Department of Molecular Biology and Biophysics, UConnHealth, Farmington, CT, 06032, USA; Constantine Petridis: Massachusetts Institute of Technology, Department of Biology, Cambridge, MA, 02139, USA**.**

Contains

Supporting figures S1-S7 (pages S-2 to S-9)

Extended experimental procedures (pages S-10 to S-14)

Supporting tables S1-S4 (pages S-15 to S-38)

Supporting figures

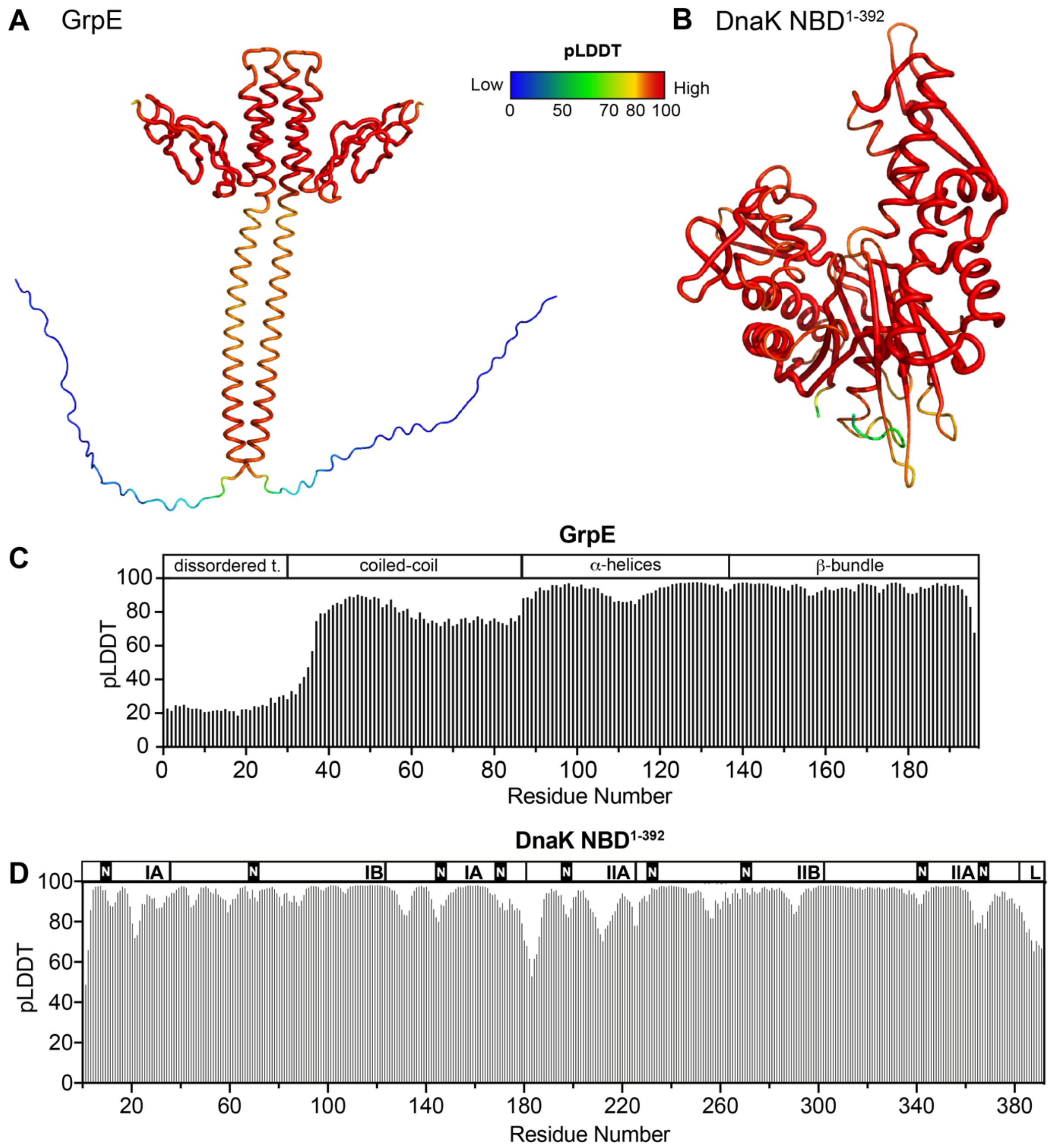

Figure S1. Estimated confidence per-residue (pLDDT) for the AlphaFold-predicted structure of the NBD$^{1\text{-}392}$/GrpE complex. pLDDT were mapped in the predicted structure of the NBD$^{1\text{-}392}$/GrpE complex and shown as individual proteins for simplicity in (**A**) GrpE, and (**B**) NBD. pLDDTs are shown in a color and thickness scale, where red and thick lines indicate the highest confidence. Histograms of the pLDDT values for the amide backbone N *vs* residue number of **(C)** GrpE and **(D)** NBD.

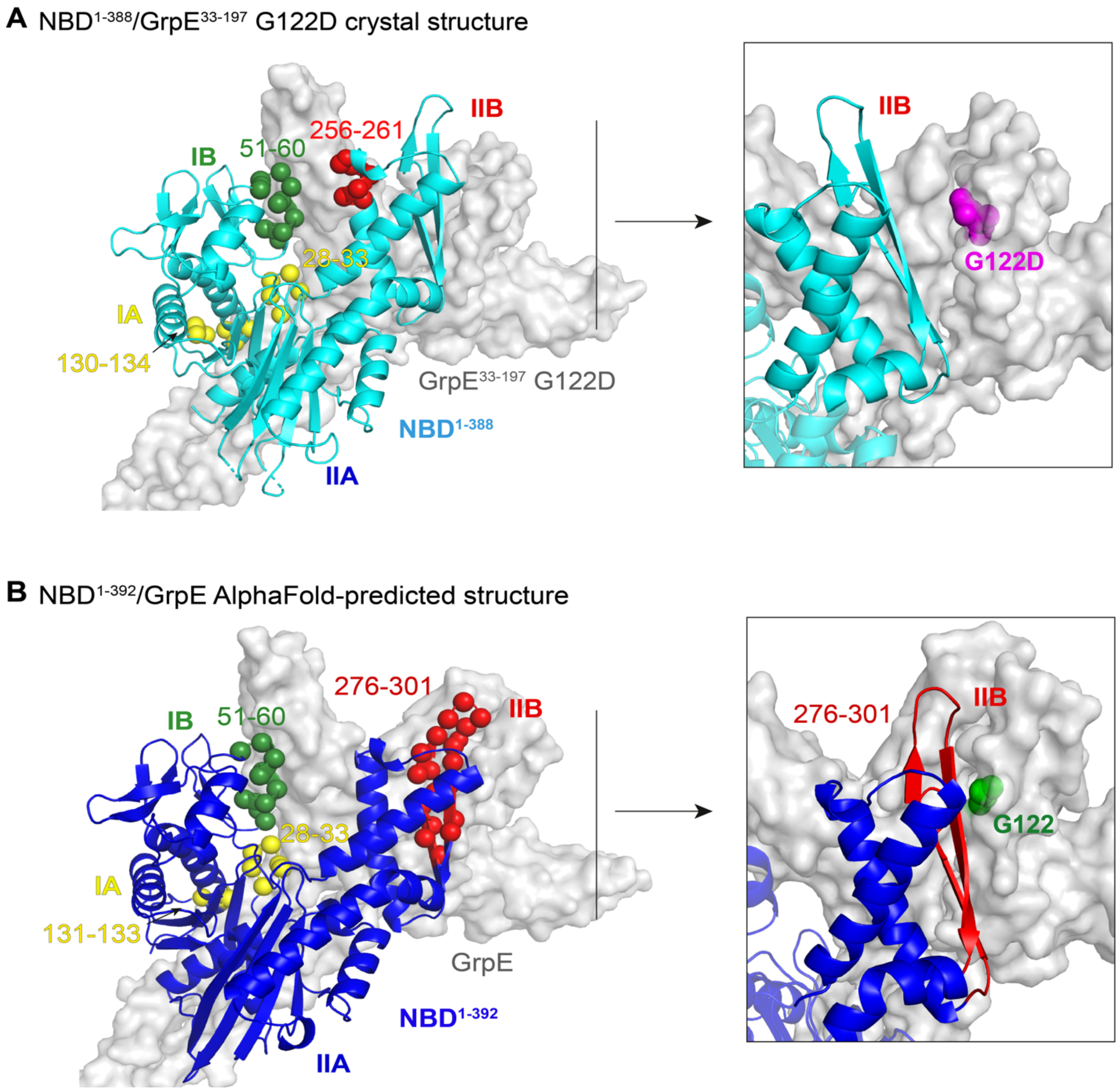

Figure S2. Interaction interfaces between NBD and GrpE in the complex. Residues that participate in the interaction interfaces are shown in the NBD as spheres, colored depending on subdomain as in Figure 1. A zoom in subdomain IIB is shown to depict the position of G122D mutation. **(A)** Crystal structure of $NBD^{1\text{-}388}$/$GrpE^{33\text{-}197}$ G122D (PDB 1dkg), **(B)** AlphaFold-predicted structure of $NBD^{1\text{-}392}$/GrpE.

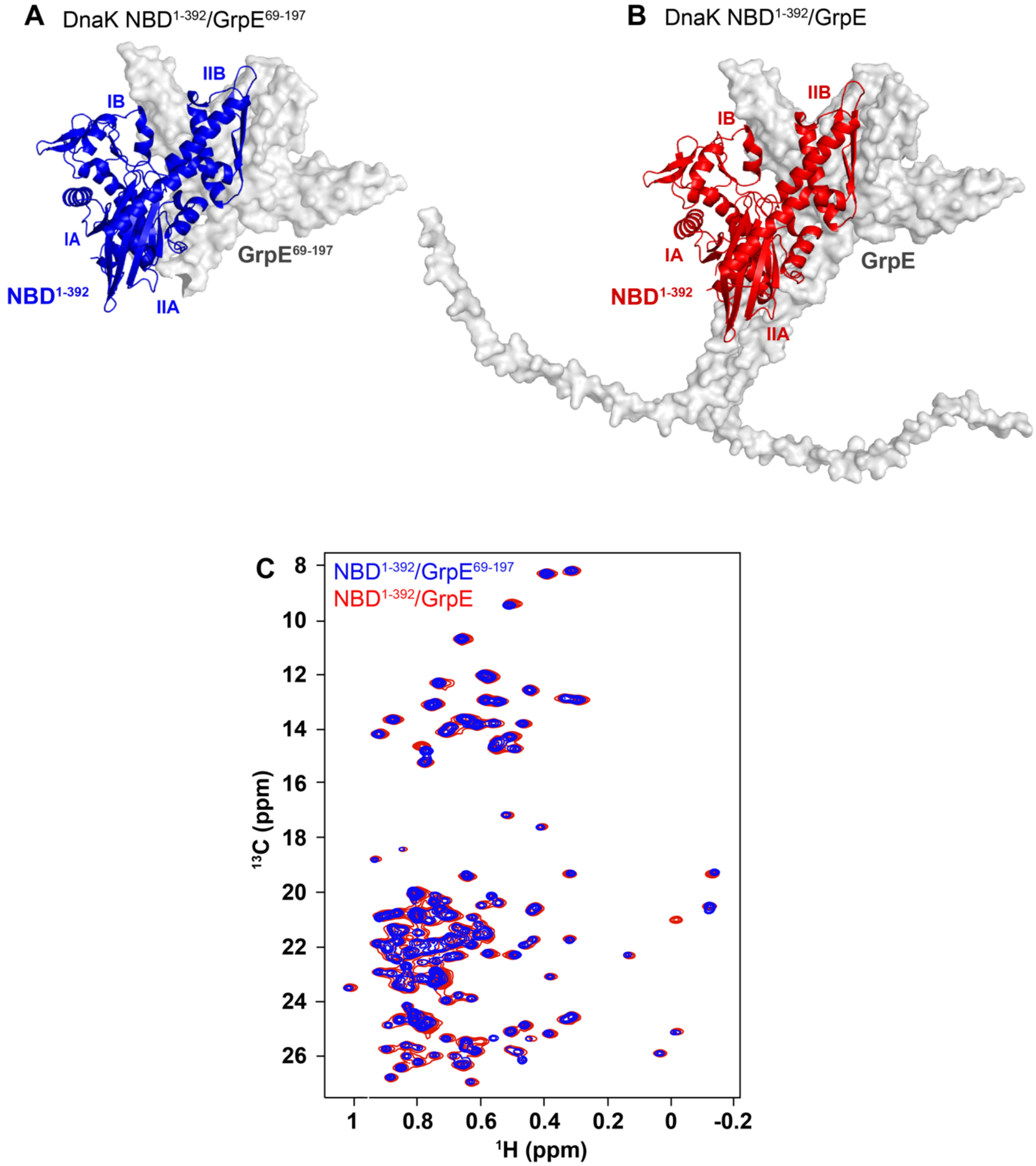

Figure S3. GrpE$^{69-197}$ forms complexes with NBD$^{1-392}$ as wild-type GrpE. AlphaFold-predicted structures of the DnaK NBD$^{1-392}$ in complex with **(A)** GrpE$^{69-197}$, and **(B)** GrpE. **(C)** ^{1}H-^{13}C HMQC spectra of ILV ^{13}C-methyl-labelled NBD$^{1-392}$ in complex with GrpE, or GrpE$^{69-197}$. For the chemical shift data see Table S2.

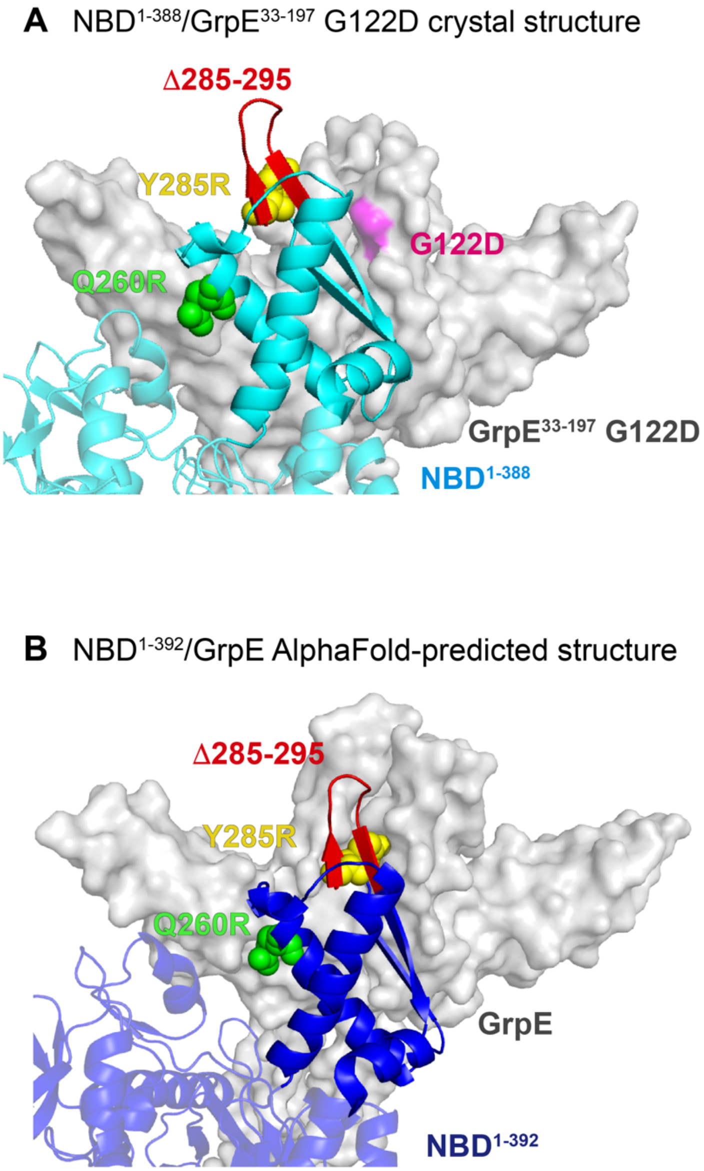

Figure S4. Substitutions in subdomain IIB of DnaK NBD$^{1\text{-}392}$ designed to experimentally validate the AlphaFold-predicted structure of NBD/GrpE. Designed mutations are mapped in **(A)** the crystal structure of NBD$^{1\text{-}388}$/GrpE$^{33\text{-}197}$ G122D (PDB 1dkg), and **(B)** in the AlphaFold-predicted structure of NBD$^{1\text{-}392}$/GrpE.

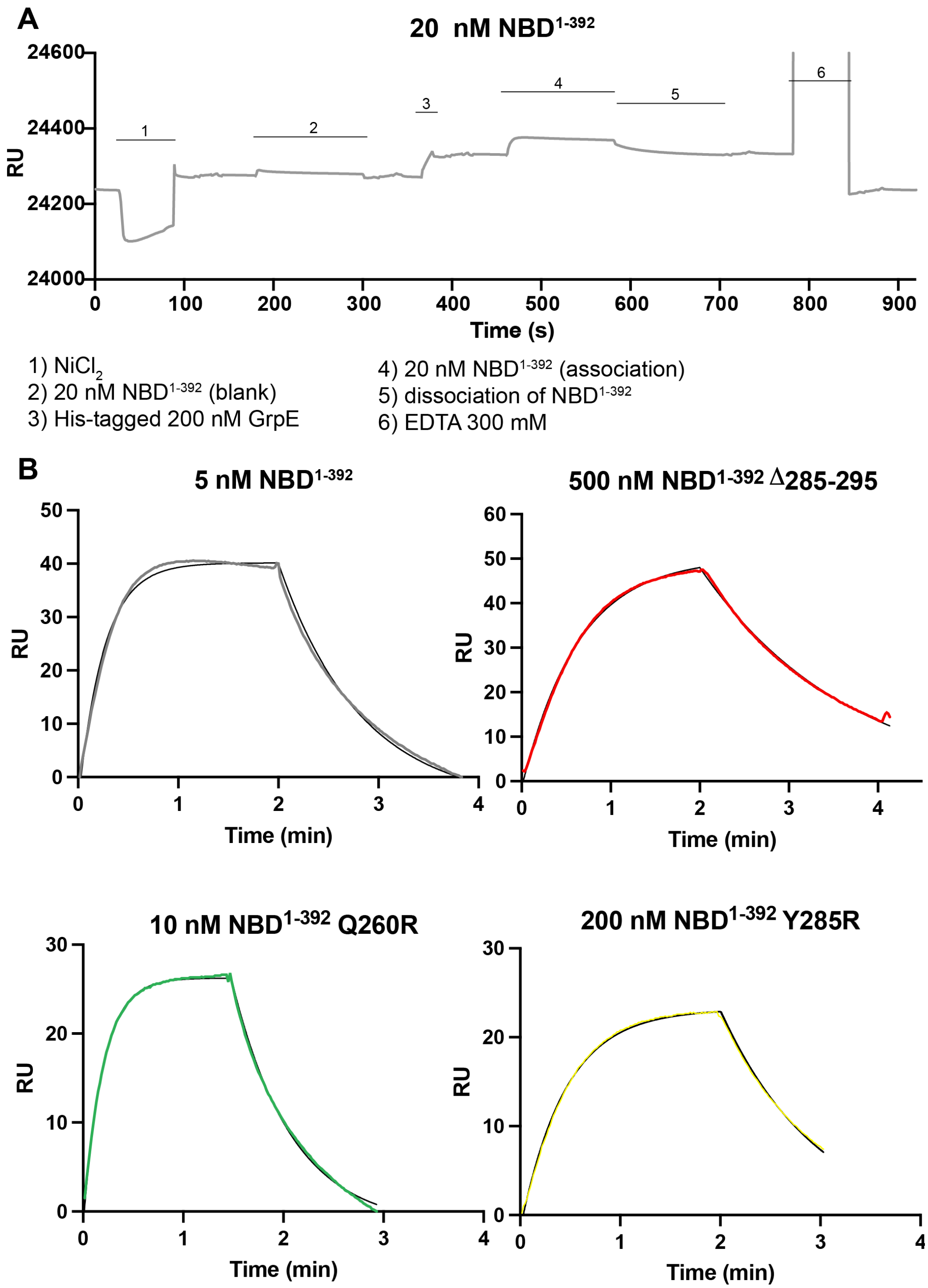

Figure S5. SPR sensorgrams and curve fitting. (A) Representative sensorgram for the K_D determinations of NBD$^{1\text{-}392}$ (or its variants) binding to His-tagged GrpE. **(B)** Examples of processed binding curves (color lines) and their fitting (black lines) to calculate the K_D, k_{on}, and k_{off}. Each curve shows the binding of the indicated NBD$^{1\text{-}392}$ variant to His$_6$-GrpE immobilized on a Ni-NTA surface.

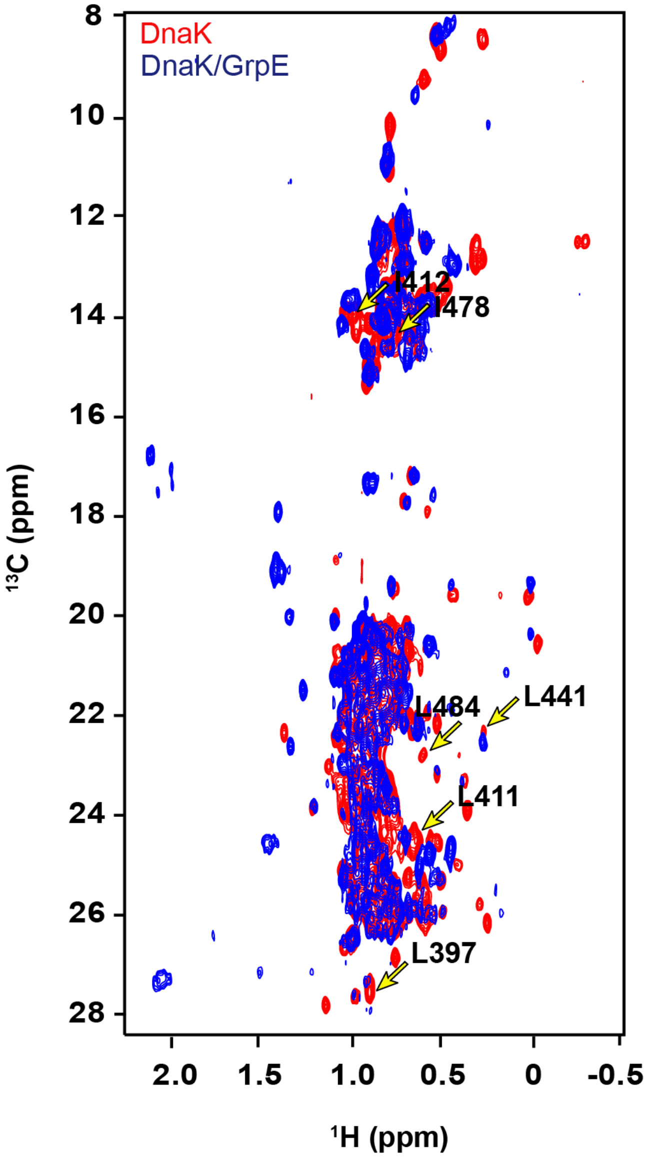

Figure S6. NMR-signals corresponding to the DnaK SBD show perturbations upon GrpE binding to DnaK. ^{1}H-^{13}C-HMQC spectra of ILV ^{13}C-methyl-labelled DnaK alone and in complex with GrpE. Yellow arrows indicate resonances of DnaK SBD that are shifted upon GrpE binding. For the chemical shift data see Table S1.

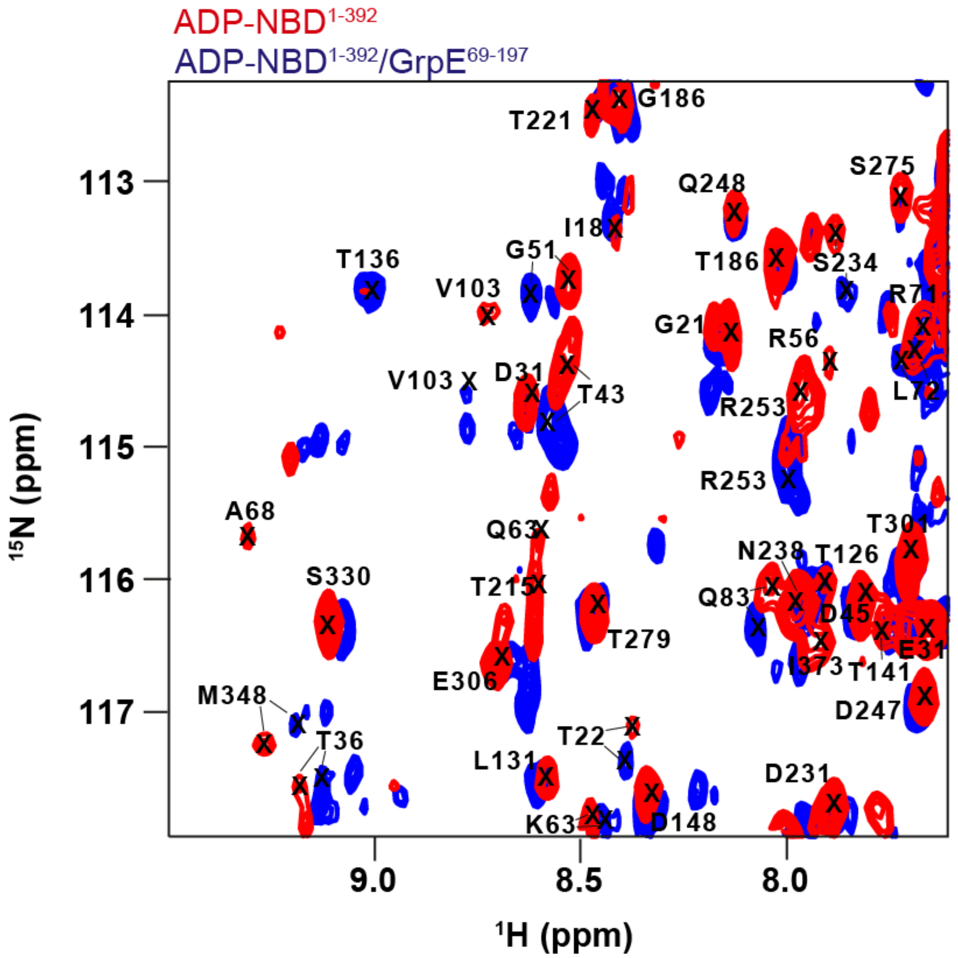

Figure S7. Zoomed-in region of the ^{1}H-^{15}N HSQCs of ADP-^{15}N NBD$^{1\text{-}392}$ and ADP-^{15}N NBD$^{1\text{-}392}$/GrpE$^{69\text{-}197}$ complex.

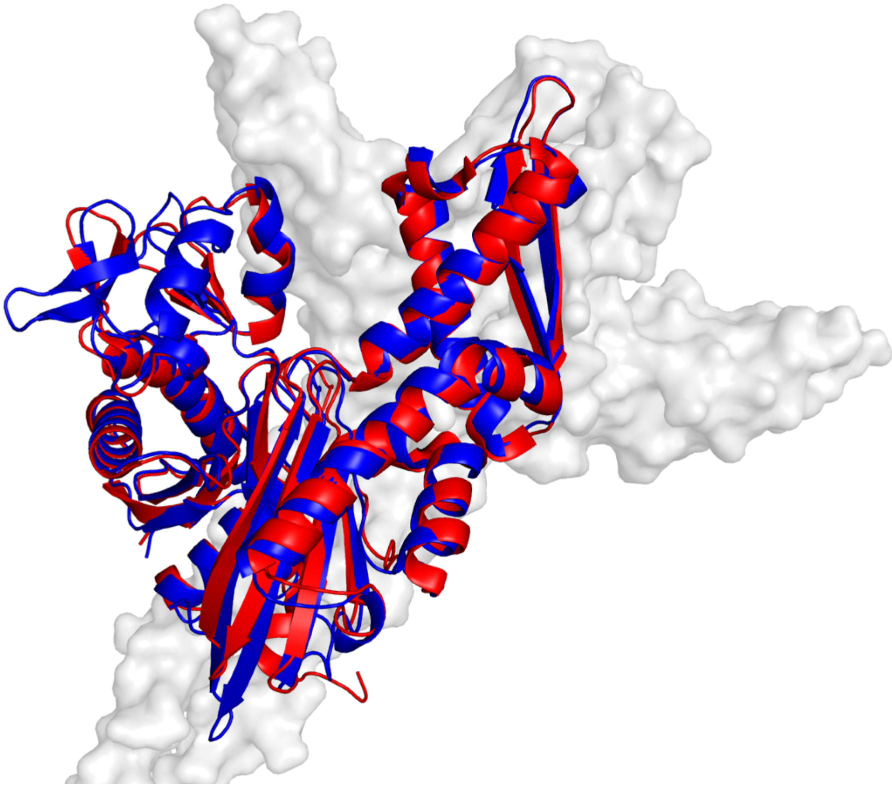

Figure S8. AlphaFold-predicted complex is close to the *Geobacillus kaustophilus* DnaK/GrpE crystallized one. Overlay of AlphaFold-predicted structure of the DnaK NBD$^{1\text{-}392}$/GrpE complex (NBD in blue, GrpE in grey) with the NBD (red) from *Geobacillus kaustophilus* DnaK/GrpE complex (PDB 4ani).

Extended experimental procedures

Plasmids

pMKS plasmid (48) was used to express *E. coli* full-length DnaK (1-638), and pMS was used to express DnaK NBD$^{1\text{-}392}$ T199A (37). NBD variants were obtained by site-directed mutagenesis.

The plasmid pMCSG7 containing the *E. coli* GrpE gene (pMCSgrpe) was a generous gift from Jason E. Gestwicki (University of California San Francisco) (17). The plasmids harboring GrpE$^{69\text{-}197}$ and GrpE$^{69\text{-}197}$ G122D-encoding genes were obtained by site-directed mutagenesis.

Protein expression and purification

For DnaK, DnaK NBD$^{1\text{-}392}$ T199A, and its variants, *E. coli* BL21(*DE3*) carrying the appropriate plasmid were grown in LB supplemented with 100 μg/mL ampicillin at 37 °C until an OD_{600} 0.6-0.9. Protein expression was induced by addition of 0.2 mM IPTG and cells were incubated for an additional 4 h at the same temperature. All subsequent steps were done at 4 °C. Cells were harvested by centrifugation, resuspended in lysis buffer (20 mM Hepes, 50 mM NaCl and 0.1 mM EDTA, 1 mM phenylmethylsulfonyl fluoride (PMSF), pH 8.0) and lysed by sonication. The lysate was cleared by centrifugation 48,300 xg at 4 °C for 15 min. 100 mg streptomycin sulfate were added to the cleared lysate followed by centrifugation 48,300 xg at 4 °C for 15 min. The soluble fraction was loaded in a DEAE Sepharose anion exchange column (HiPrep DEAE FF 16/10, Cytiva # 28936541) equilibrated with buffer A (20 mM Hepes, 1 mM EDTA, pH 7.4). Proteins were eluted with a gradient (0-100%, 10 column volumes) of buffer B (20 mM Hepes, 1 mM EDTA, 1 M KCl, pH 7.4). The fractions containing DnaK, NBD$^{1\text{-}392}$ T199A or NBD$^{1\text{-}392}$ variants were identified by SDS-PAGE, pooled, supplemented with $MgCl_2$ to 10 mM, and loaded into an ATP-agarose column (C8-linked ATP resin, Sigma A2767) equilibrated with buffer C (20 mM Hepes, 10 mM $MgCl_2$, 100 mM NaCl, pH 7.6). The column was washed with buffer C supplemented with 2 M KCl, and proteins were eluted with buffer C supplemented with 2 mM ADP. To obtain nucleotide-free NBD$^{1\text{-}392}$ T199A, 2 U of phosphatase alkaline (calf intestine, Millipore/Sigma, 524572) were added per 1 mg of NBD, and incubated 1 h at room temperature, followed by a dialysis against buffer C at 4 °C for 16 h. Phosphatase alkaline was remove by size exclusion

chromatography (HiLoad 16/600, Superdex 200, Cytiva, 28-9873-35) in 20 mM Hepes, 100 mM KCl, pH 7.6. To obtain nucleotide-free DnaK, the protein was unfolded in 20 mM Hepes, 8 M urea, pH 7.6 for 2 h. The solution was concentrated by ultrafiltration using centrifugal devices (Millipore/Sigma Amicon Ultra-15 Centrifugal Filter Units, 30 MWCO), and DnaK in urea buffer was added by dropwise dilution into 20X buffer C at 4 °C with slow agitation. Under these conditions, refolding was allowed to proceed for 4 h. The purity of the nucleotide-free proteins was determined by SDS-PAGE and their concentrations by UV spectroscopy using the molar extinction coefficients of 15800 $cm^{-1}M^{-1}$ for NBD and 15930 $cm^{-1}M^{-1}$ for DnaK. Proteins were flash frozen and stored at -80 °C.

For expression of GrpE and its variants, *E. coli* BL21(*DE3*) carrying the appropriate plasmids were grown in LB supplemented with 100 μg/mL ampicillin at 37 °C until an OD_{600} was 0.6-0.9. Protein expression was induced by addition of 0.2 mM IPTG, and cells were incubated for additional 4 h at the same temperature. All subsequent steps were done at 4 °C. Cells were harvested by centrifugation, resuspended in lysis buffer 2 (50 mM Tris, 100 mM NaCl, 20 mM imidazole, 1 mM PMSF, pH 8.0) and lysed by sonication. The lysate was cleared by two rounds of centrifugation 48,300 xg at 4 °C for 30 min, with the addition of 100 mg streptomycin sulfate in between. The soluble fraction was loaded in a Ni-NTA column (HisTrap FF, Cytiva # 17531901) equilibrated with buffer D (50 mM Tris, 100 mM NaCl, 20 mM imidazole, pH 8). The column was washed with 5 column volumes of buffer D and then 5 column volumes of buffer D with 2 mM ATP. The proteins were eluted with a gradient (0-100%, 10 column volumes) of buffer D with 500 mM imidazole. The fractions containing His_6-tagged GrpE, or its variants, were treated overnight with TEV protease (1 mg_{TEV}:100 mg_{GrpE}) in a dialysis against buffer D with 10 mM β-mercaptoethanol. The digestion mixture was passed through the Ni-NTA column again to remove the undigested protein, digested His_6, and His_6-tagged TEV. The purity of the protein was determined by SDS-PAGE and the concentration by UV spectroscopy using a molar extinction coefficient of 1480 $cm^{-1}M^{-1}$ for GrpE. Proteins were flash frozen and stored at -80 °C. GrpE concentrations in this manuscript always refer to the dimer. It has previously been shown that $GrpE^{69-197}$ is a dimer. (39)

To prepare uniformly ^{2}H-^{15}N-labeled NBD$^{1-392}$ T199A or ^{2}H-labeled GrpE$^{69-197}$ the cells were grown in M9 minimal medium in D_2O containing $^{15}NH_4Cl$ (Cambridge Isotope Laboratories, Inc., NLM-467), if appropriate, using the established protocol.(49) After addition of 0.2 mM IPTG, cells were allowed to grow for 16 h at 20 °C. To prepare DnaK and NBD$^{1-392}$ T199A with selectively labeled Ile, Leu and Val methyl groups (i.e., [U-^{2}H,^{12}C,^{15}N]; Ileδ1-[^{13}CH3]; Leu, Val-[$^{13}CH_3$,$^{12}CD_3$]), cells were grown in M9 minimal medium in D_2O containing D-glucose (D-glucose (1,2,3,4,5,6,6-D7, 97-98%), Cambridge Isotope Laboratories, Inc, DLM-2062). The same protocol was used for bacterial growth and additionally, sodium salts of α-ketobutyric (methyl-^{13}C, 3,3-D2, Cambridge Isotope Laboratories, CLM-6820) and α-ketoisovaleric (3-methyl-^{13}C, 3,4,4,4-D4, Cambridge Isotope Laboratories, CDLM-7371) acids were added (70 mg/L and 120 mg/L, respectively) 1 h before induction. The proteins were purified following the method described above.

NMR spectroscopy

NMR experiments were carried out at 25 °C on a 600 MHz Bruker Avance III spectrometer equipped with a CryoProbe. Data were processed with TopSpin 3.6.2. and analyzed in CcpNmr Analysis Version 3 AnalysisAssign (29, 52). For ^{15}N-TROSY experiments the NMR buffer was 10 mM potassium phosphate, 5 mM DTT, 0.02% sodium azide, 0.1 mM 4-benzenesulfonyl fluoride hydrochloride (AEBSF), 10 % D_2O, pH 7.4. HSQCs spectra were acquired with the pulse sequence trosyetfpf3gpsi, non-uniform sampling detection (50 %, Poisson-gap sampling method (53)), number of points TD_{1H} 2048 and TD_{15N} 200. The protein concentrations, conditions, and number of scans (NS) of each experiment were the following:

- 300 μM ^{2}H-^{15}N NBD$^{1-392}$ T199A, NS 64
- 300 μM ^{2}H-^{15}N NBD$^{1-392}$ T199A + 300 μM ^{2}H GrpE$^{69-197}$, NS 64
- 300 μM ^{2}H-^{15}N NBD$^{1-392}$ T199A + 450 μM ^{2}H GrpE$^{69-197}$ G122D, NS 200
- 300 μM ^{2}H-^{15}N ADP-NBD$^{1-392}$ T199A + 3 mM ADP-Mg, NS 64
- 300 μM ^{2}H-^{15}N ADP-NBD$^{1-392}$ T199A + 3 mM ADP-Mg + 600 μM ^{2}H GrpE$^{69-197}$, NS 124.

For ILV ^{13}C-labeled experiments the NMR buffer was 10 mM potassium phosphate, 5 mM DTT, 0.02% sodium azide, 0.1 mM AEBSF, 100 % D_2O, pD 7.4. HMQCs spectra

were acquired with the pulse sequence hmqcphpr, number of points TD_{1H} 2048 and TD_{13C} 256. The protein concentrations, conditions, and number of scans (NS) of each experiment were the following:

- 40 μM ^{2}H-^{13}C ILV NBD$^{1-392}$ T199A, NS 16
- 40 μM ^{2}H-^{13}C ILV NBD$^{1-392}$ T199A + 40 μM GrpE$^{69-197}$, NS 32
- 40 μM ^{2}H-^{13}C ILV NBD$^{1-392}$ T199A + 40 μM GrpE$^{33-197}$, NS 32
- 40 μM ^{2}H-^{13}C ILV NBD$^{1-392}$ T199A + 40 μM GrpE, NS 32
- 40 μM ^{2}H-^{13}C ILV ADP-NBD$^{1-392}$ T199A + 400 μM ADP-Mg, NS 16
- 40 μM ^{2}H-^{13}C ILV ADP-NBD$^{1-392}$ T199A + 400 μM ADP-Mg + 80 μM GrpE$^{69-197}$, NS 32
- 40 μM ^{2}H-^{13}C ILV ADP-NBD$^{1-392}$ T199A + 400 μM ADP-Mg + 80 μM GrpE$^{33-197}$, NS 32
- 40 μM ^{2}H-^{13}C ILV ADP-NBD$^{1-392}$ T199A + 400 μM ADP-Mg + 80 μM GrpE, NS 32
- 40 μM ^{2}H-^{13}C ILV DnaK$^{1-638}$, NS 32
- 60 μM ^{2}H-^{13}C ILV DnaK$^{1-638}$ + 80 μM GrpE$^{69-197}$, NS 64
- 60 μM ^{2}H-^{13}C ILV DnaK$^{1-638}$ + 80 μM GrpE$^{33-197}$, NS 64
- 60 μM ^{2}H-^{13}C ILV DnaK$^{1-638}$ + 80 μM GrpE, NS 64

Surface Plasmon Resonance (SPR) measurements

SPR measurements were done in a Biacore T-200 instrument with NTA sensor chips (Serie S sensor chip NTA, Cytiva, BR100532). The running buffer was 10 mM Hepes, 150 mM KCl, 0.05 % P20, pH 7.6. In each sensorgram, the sensor chip was first activated with Ni^{2+} (0.5 mM $NiCl_2$, contact time 60 s, flow rate 10 μL/min), which typically produce a response (RU) of ~ 80. N-terminal His_6-GrpE was immobilized (200 nM His_6-GrpE, contact time 15 s, flow rate 10 μL/min) with a RU ~100. NBD$^{1-392}$ T199A, or its variants, were perfused (contact time 120 s, flow rate 40 μL/min, dissociation time 120 s) at diverse concentrations depending on the K_D. Finally, the sensor chip was regenerated with a metal chelating agent (300 mM EDTA, contact time 60 s, flow rate 30 μL/min) and extensively washed with running buffer. The control sensorgram were generated by following the described protocol but omitting the His_6-GrpE immobilization step. All proteins were extensively dialyzed against the running buffer prior to the

measurements. Each binding data represents the sensorgram minus the control and were analyzed with GraphPad Prism 9.

Each data set was fitted with two different methods, 1) the RU at binding equilibrium (RU_{eq}) were measured at each NBD, or its variants, concentrations, and were fitted with the hyperbola function $RU_{eq} = (RU_{max}*[NBD])/(K_D+[NBD])$; 2) the association and dissociation of each sensorgram was fitted according with the equations,

$$\textit{Association} \quad RU_a = \frac{RU_{max} \times [NBD]}{K_D + [NBD]} \left(1 - e^{-k_{obs} \times t}\right), \quad k_{obs} = [NBD]\, k_{on} + k_{off}$$

$$\textit{Dissociation} \quad RU_d = RU_0\, e^{-k_{off} \times (t - t_0)}$$

Table S1. Chemical shifts of ^{1}H-^{13}C ILV methyl labelled apoDnaK and in complex with GrpE.

Table S1. Chemical shifts of ^{1}H-^{13}C ILV methyl labelled-apoDnaK and in complex with GrpE.

Residue	$-CH_3$	apoDnaK		apoDnaK/GrpE	
		^{1}H (ppm)	^{13}C (ppm)	^{1}H (ppm)	^{13}C (ppm)
Ile4	Hg2	0.82	13.94	0.82	14.05
Ile5	Hd1	0.71	13.92	0.71	13.92
Ile7	Hd1	1.02	14.02	1.01	13.79
Val16	Hg1	1.08	18.98	1.06	18.90
Val16	Hg2	0.90	22.95	0.90	22.95
Ile18	Hd1	0.90	15.09	0.92	14.73
Val26	Hg1	0.89	22.64	0.89	22.64
Leu27	Hd1	-0.04	20.67	0.00	20.47
Ile39	Hg2	0.80	13.52	0.88	13.55
Ile40	Hd1	0.66	17.30	0.65	17.29
Leu49	Hd1	0.67	22.21	0.63	22.22
Leu49	Hd2	0.61	25.40	0.62	25.12
Leu66	Hd1	0.35	24.02	0.44	24.73
Ile69	Hg2	0.50	8.72	0.46	8.29
Ile73	Hd1	0.71	12.39	0.71	12.24
Val86	Hg1	1.04	22.15	1.05	22.02
Val86	Hg2	1.02	20.93	1.02	20.94
Ile88	Hd1	0.87	13.25	0.88	13.26
Ile93	Hd1	0.80	14.23	0.80	14.21
Ile94	Hd1	0.74	13.86	0.74	13.93
Val103	Hg1	0.69	20.29	0.67	20.37
Val105	Hg1	0.73	20.62	0.72	20.59
Ile115	Hd1	0.59	12.62	0.58	12.60
Leu131	Hd1	1.04	22.69	1.05	23.06
Leu131	Hd2	0.83	25.46	0.82	25.41
Val139	Hg1	0.75	20.76	0.79	20.95
Val139	Hg2	0.77	20.34	0.77	20.53
Ile140	Hg2	0.67	14.44	0.67	14.55
Val142	Hg1	0.57	18.01	0.54	17.66
Ile160	Hd1	0.57	13.80	0.56	13.87
Leu163	Hd1	0.52	23.28	0.52	23.20
Leu163	Hd2	0.49	25.41	0.51	25.33
Val165	Hg1	0.76	21.29	0.76	21.29
Ile168	Hd1	0.75	12.41	0.68	13.05
Ile169	Hg2	0.46	13.49	0.44	13.02
Leu177	Hd1	0.51	24.65	0.56	24.84
Leu177	Hd2	0.64	22.37	0.62	22.42
Leu181	Hd1	0.74	21.99	0.75	21.92

Table S1. Chemical shifts of ^{1}H-^{13}C ILV methyl labelled apoDnaK and in complex with GrpE.

Ile190	Hd1	0.62	14.79	0.62	14.82
Val192	Hg1	0.74	19.55	0.78	19.53
Ile202	Hd1	1.04	14.05	1.04	14.26
Ile204	Hg2	0.78	10.25	0.79	10.79
Ile205	Hd1	0.71	12.97	0.71	13.02
Val210	Hg1	0.84	20.71	0.84	20.60
Leu219	Hd1	0.97	25.56	0.97	25.62
Leu227	Hd2	0.66	24.67	0.70	24.54
Ile237	Hd1	0.90	14.24	0.87	14.21
Leu240	Hd1	1.01	23.98	0.98	24.00
Leu240	Hd2	0.98	25.78	1.00	25.92
Ile250	Hd1	0.77	13.68	0.77	13.76
Leu252	Hd1	0.52	22.26	0.44	21.98
Leu252	Hg,	0.24	26.27	0.17	26.06
Leu257	Hd1	1.00	24.24	0.95	24.24
Leu262	Hd1	0.73	26.13	0.73	26.00
Leu262	Hd2	0.76	25.49	0.77	25.53
Leu273	Hd1	0.57	22.03	0.56	21.93
Leu283	Hd1	0.76	26.21	0.76	26.21
Ile286	Hd1	0.91	15.46	0.89	15.27
Ile298	Hd1	0.61	14.63	0.60	14.34
Val309	Hg1	0.88	18.52	0.87	17.47
Leu312	Hd1	0.28	25.89	0.19	25.61
Ile317	Hd1	0.53	8.48	0.52	8.48
Leu324	Hd1	0.99	26.56	0.98	26.57
Leu324	Hd2	0.78	23.70	0.80	23.55
Leu324	Hg,	1.14	27.90	1.21	27.25
Leu329	Hd2	0.75	26.94	0.75	27.08
Val331	Hg1	0.79	20.09	0.78	20.09
Val331	Hg2	0.71	22.44	0.70	22.29
Ile334	Hd1	0.59	9.34	0.64	9.68
Val337	Hg1	0.01	19.71	0.00	19.47
Val337	Hg2	0.42	19.69	0.44	19.48
Ile338	Hd1	0.30	12.65	0.42	13.10
Leu339	Hd1	0.91	26.44	0.90	26.39
Val349	Hg1	1.12	23.12	1.00	23.06
Val353	Hg2	0.10	21.54	0.13	21.27
Val365	Hg1	0.61	21.14	0.56	20.69
Ile373	Hd1	0.69	13.93	0.69	13.93
Val381	Hg1	1.07	20.85	1.05	21.27
Leu397	Hd1	1.21	23.95	1.20	23.92
Leu399	Hd1	1.03	26.76	1.03	26.63
Ile401	Hd1	-0.31	12.59	1.33	11.40

Table S1. Chemical shifts of ^1H-^{13}C ILV methyl labelled apoDnaK and in complex with GrpE.

Leu411	Hd1	0.63	24.61	0.68	24.57
Ile412	Hd1	0.98	14.04	0.98	13.74
Ile418	Hd1	0.74	13.45	0.74	13.63
Ile438	Hd1	0.26	8.54	0.24	10.26
Leu454	Hd1	0.68	26.05	0.67	25.99
Leu459	Hd1	0.75	26.47	0.77	26.42
Ile462	Hd1	0.55	13.77	0.54	13.89
Ile472	Hd1	0.68	12.39	0.67	12.25
Ile478	Hd1	0.78	14.48	0.78	14.73
Ile483	Hd1	0.78	11.16	0.80	11.09
Leu484	Hd1	0.59	22.89	1.70	22.64
Ile499	Hd1	0.79	13.61	0.79	13.69
Ile501	Hd1	0.60	13.86	0.60	13.91
Leu507	Hd1	0.74	21.61	0.73	21.59
Ile512	Hd1	0.81	13.70	0.81	13.67
Leu532	Hd1	0.95	23.81	0.95	23.81
Ile565	Hd1	0.87	15.11	0.85	15.27
Val394	Hg1	0.70	17.80	0.68	17.84
Leu397	Hd2	0.89	27.66	0.91	27.45
Leu441	Hd1	0.25	22.68	0.27	22.64
Val486	Hg1	0.17	19.69	0.17	19.60
Val516	Hg1	1.08	22.52	1.07	22.44

Table S2. Chemical shifts of ^{1}H-^{13}C ILV methyl labelled apoNBD$^{1-392}$ and in complex with GrpE and GrpE$^{69-197}$.

Table S2. Chemical shifts of ^{1}H-^{13}C ILV methyl labelled apoNBD$^{1-392}$ and in complex with GrpE and GrpE$^{69-197}$.

Residue	-CH$_3$	apoNBD$^{1-392}$		apoNBD$^{1-392}$/GrpE		apoNBD$^{1-392}$/GrpE$^{69-197}$	
		^{1}H (ppm)	^{13}C (ppm)	^{1}H (ppm)	^{13}C (ppm)	^{1}H (ppm)	^{13}C (ppm)
Ile4	Hg2	0.70	14.17	0.71	14.19	0.71	14.17
Ile5	Hd1	0.56	13.87	0.57	13.87	0.56	13.85
Ile7	Hd1	0.92	13.98	0.88	13.72	0.87	13.72
Val16	Hg1	0.94	18.90	0.93	18.88	0.93	18.87
Val16	Hg2	0.79	22.59	0.78	22.90	0.83	22.79
Ile18	Hd1	0.77	15.01	0.79	14.69	0.77	14.86
Val26	Hg1	0.75	22.46	0.75	22.58	0.74	22.58
Leu27	Hd1	-0.17	20.66	-0.12	20.58	-0.12	20.57
Ile39	Hg2	0.64	13.41	0.64	13.70	0.64	13.70
Ile40	Hd1	0.52	17.21	0.52	17.23	0.52	17.21
Leu49	Hd1	0.59	21.96	0.63	21.94	0.63	21.99
Leu49	Hd2	0.45	25.88	0.48	25.95	0.48	25.93
Val50	Hg1	0.75	22.08	-	-	-	-
Val59	Hg1	0.82	20.88	0.80	20.84	0.80	20.83
Leu66	Hd2	0.51	25.39	0.51	25.18	0.50	25.16
Leu66	Hg	0.26	24.06	0.31	24.64	0.31	24.63
Ile69	Hg2	0.34	8.56	0.31	8.23	0.31	8.25
Ile73	Hd1	0.57	12.08	0.57	12.19	0.57	12.19
Val86	Hg1	0.92	21.97	0.92	21.95	0.92	21.94
Val86	Hg2	0.89	20.86	0.89	20.89	0.89	20.89
Ile88	Hd1	0.75	13.15	0.75	13.18	0.76	13.19
Ile93	Hd1	0.69	13.96	0.69	13.99	0.70	13.99
Ile94	Hd1	0.62	13.95	0.61	13.91	0.61	13.91
Val103	Hg1	0.56	20.35	0.57	20.27	0.56	20.19
Val105	Hg1	0.52	20.63	0.54	20.45	0.54	20.45
Val105	Hg2	0.67	21.23	0.67	21.43	0.67	21.34
Ile115	Hd1	0.45	12.61	0.44	12.64	0.45	12.64
Leu131	Hd1	0.89	22.75	0.89	23.04	0.88	23.02
Leu131	Hd2	0.71	25.52	0.71	25.40	0.71	25.44
Val139	Hg2	0.60	20.57	0.59	20.51	0.60	20.54
Ile140	Hg2	0.54	14.38	0.51	14.34	0.51	14.35
Val142	Hg1	0.41	17.66	0.40	17.68	0.41	17.68
Val142	Hg2	0.78	23.23	0.79	23.26	0.78	23.22
Ile160	Hd1	0.47	13.81	0.46	13.86	0.47	13.87
Leu163	Hd1	0.38	23.14	0.38	23.17	0.38	23.16
Leu163	Hd2	0.37	25.08	0.38	25.26	0.38	25.24

Table S2. Chemical shifts of ^{1}H-^{13}C ILV methyl labelled apoNBD$^{1-392}$ and in complex with GrpE and GrpE$^{69-197}$.

Val165	Hg1	0.63	21.38	0.59	21.54	0.58	21.59
Ile168	Hd1	0.60	12.23	0.58	12.12	0.59	12.09
Ile169	Hg2	0.29	12.76	0.33	12.95	0.33	12.94
Leu177	Hd1	0.42	24.94	0.46	24.91	0.46	24.93
Leu177	Hd2	0.57	22.37	0.57	22.31	0.58	22.30
Leu181	Hd1	0.63	21.81	0.61	21.60	0.61	21.62
Ile190	Hd1	0.54	14.52	0.55	14.68	0.55	14.71
Val192	Hg1	0.63	19.68	0.64	19.41	0.64	19.42
Ile202	Hd1	0.91	14.35	0.92	14.24	0.92	14.25
Ile204	Hg2	0.64	10.53	0.66	10.77	0.66	10.75
Ile205	Hd1	0.54	13.22	0.55	13.06	0.54	13.06
Ile207	Hd1	0.72	12.50	0.73	12.38	0.73	12.36
Val210	Hg1	0.72	20.58	0.73	20.68	0.73	20.68
Leu219	Hd1	0.85	25.54	0.83	25.70	0.83	25.67
Leu219	Hd2	0.72	21.00	0.70	20.90	0.70	20.91
Leu227	Hd2	0.50	24.80	-	-	-	-
Leu240	Hd1	0.86	23.64	0.85	23.43	0.85	23.46
Leu240	Hd2	0.88	25.59	0.90	25.81	0.90	25.81
Ile250	Hd1	0.65	13.67	0.64	13.70	0.65	13.70
Leu252	Hd1	0.39	22.12	0.32	21.79	0.32	21.82
Leu252	Hg	0.09	26.28	0.03	26.00	0.03	25.97
Leu257	Hd1	0.85	24.03	-	-	-	-
Leu262	Hd1	0.61	25.84	0.61	25.89	0.62	25.88
Leu262	Hd2	0.63	25.70	0.65	25.54	0.64	25.55
Leu273	Hd1	0.42	22.03	0.45	21.84	0.43	21.79
Leu283	Hd1	0.64	26.25	0.65	26.41	0.65	26.39
Ile286	Hd1	0.77	15.07	0.78	15.30	0.78	15.28
Ile298	Hd1	0.47	14.66	0.50	14.79	0.49	14.80
Val309	Hg1	0.86	18.25	0.85	18.48	0.85	18.48
Leu312	Hd1	-0.04	25.31	-0.01	25.21	-0.01	25.20
Ile317	Hd1	0.40	8.38	0.39	8.37	0.39	8.36
Leu324	Hd1	0.84	26.45	0.85	26.50	0.85	26.50
Leu324	Hd2	0.66	23.75	0.67	23.85	0.67	23.84
Leu324	Hg	0.90	26.99	0.88	26.85	0.88	26.85
Leu329	Hd1	0.69	21.97	0.69	21.79	0.70	21.73
Leu329	Hd2	0.63	27.07	0.63	27.01	0.63	27.02
Val331	Hg2	0.50	22.31	0.49	22.36	0.49	22.36
Ile334	Hd1	0.50	9.41	0.51	9.53	0.51	9.52
Val337	Hg1	-0.19	19.14	-0.14	19.34	-0.14	19.33
Val337	Hg2	0.28	19.33	0.32	19.36	0.31	19.36

Table S2. Chemical shifts of ^{1}H-^{13}C ILV methyl labelled apoNBD$^{1-392}$ and in complex with GrpE and GrpE$^{69-197}$.

Ile338	Hd1	0.32	13.05	0.29	12.99	0.29	13.00
Val349	Hg1	1.01	23.48	1.01	23.55	1.02	23.58
Val349	Hg2	0.81	21.68	0.80	21.53	0.79	21.55
Val353	Hg1	0.11	22.07	0.14	22.39	0.14	22.36
Val365	Hg1	0.44	20.79	0.43	20.63	0.43	20.62
Ile373	Hd1	0.58	12.97	0.58	13.02	0.59	13.00
Val381	Cg2	0.84	20.98	0.86	20.84	-	-
Val381	Hg2	0.85	20.87	0.86	20.83	0.86	20.81
Val386	Hg1	0.80	20.23	0.80	20.12	0.81	20.08
Val386	Hg2	0.79	20.99	0.79	20.99	0.80	20.97
Leu391	Hd1	0.73	23.38	0.73	23.39	0.74	23.32
Leu392	Hd1	0.71	23.19	0.72	23.19	0.74	23.17

Table S3. Chemical shifts of ^{1}H-^{15}N apoNBD$^{1-392}$ and in complex with GrpE$^{69-197}$ and GrpE$^{69-197}$ G122D.

Table S3. Chemical shifts of ^{1}H-^{15}N apoNBD$^{1-392}$ and in complex with GrpE$^{69-197}$ and GrpE$^{69-197}$ G122D.

Residue	apoNBD$^{1-392}$		apoNBD$^{1-392}$/GrpE$^{69-197}$		apoNBD$^{1-392}$/GrpE$^{69-197}$ G122D	
	^{1}H (ppm)	^{15}N (ppm)	^{1}H (ppm)	^{15}N (ppm)	^{1}H (ppm)	^{15}N (ppm)
Ile4	8.03	122.34	7.99	122.72	7.99	122.45
Ile5	7.50	120.10	7.54	120.19	7.54	120.00
Gly6	9.00	109.67	8.94	109.41	8.97	109.54
Ile7	8.48	123.40	8.46	123.37	8.43	123.26
Asp8	8.09	124.29	8.14	124.31	8.07	124.16
Leu9	6.57	128.96	6.46	128.81	6.56	128.88
Gly10	7.38	114.02	7.36	114.00	7.37	113.92
Cys15	8.48	123.91	8.49	123.95	8.43	123.89
Val16	9.03	127.41	8.99	127.46	8.96	127.44
Ala17	8.95	128.20	8.98	127.93	8.95	128.05
Ile18	8.44	113.39	8.46	113.89	8.41	113.22
Met19	8.35	121.18	8.36	121.43	8.37	120.99
Asp20	8.32	128.35	8.33	128.03	8.30	128.18
Gly21	8.17	114.20	8.13	114.15	8.16	114.11
Thr23	7.62	119.02	7.53	118.85	7.60	118.84
Arg25	9.09	124.55	9.15	124.66	9.05	124.28
Val26	8.28	127.99	8.30	128.07	8.28	127.84
Leu27	7.90	125.86	7.97	125.85	7.95	125.86
Glu28	8.05	120.23	7.99	119.86	8.01	119.99
Asn29	8.03	118.47	8.02	118.46	8.03	118.47
Ala30	8.35	121.37	8.32	121.25	8.33	121.24
Glu31	7.62	116.35	7.72	116.57	7.67	116.36
Gly32	8.10	108.33	8.10	109.15	8.05	107.94
Asp33	7.78	119.69	7.88	119.41	7.74	119.56
Arg34	9.03	123.39	9.02	123.74	8.98	123.36
Thr35	7.37	108.88	7.37	109.22	7.36	108.97
Thr36	9.12	117.74	9.06	117.87	9.07	117.66
Ile39	8.37	127.47	8.40	128.01	8.38	127.76
Ile40	8.57	128.74	8.66	128.61	8.63	128.66
Ala41	9.32	127.35	9.35	127.20	9.33	127.24
Tyr42	7.80	121.31	7.81	121.54	7.78	121.32
Thr43	8.59	114.78	8.51	114.68	8.54	114.74
Asp45	7.84	116.30	7.72	115.99	7.87	116.37

Table S3. Chemical shifts of ^{1}H-^{15}N apoNBD$^{1-392}$ and in complex with GrpE$^{69-197}$ and GrpE$^{69-197}$ G122D.

Gly46	7.62	107.29	7.61	107.20	7.60	107.17
Glu47	7.52	120.77	7.52	120.99	7.52	120.90
Thr48	8.49	120.44	8.51	120.57	8.49	120.44
Leu49	9.09	127.17	9.04	126.46	9.05	126.69
Val50	8.48	120.64	8.55	120.34	8.49	120.55
Gly51	8.62	113.91	8.92	114.79	8.66	113.98
Gln52	10.48	129.13	10.37	127.19	10.39	128.05
Ala54	6.40	116.63	6.49	116.26	6.40	116.28
Lys55	8.12	122.27	8.24	124.06	8.12	122.37
Arg56	8.01	115.40	7.92	116.20	7.92	116.25
Gln57	7.01	111.47	7.02	113.44	7.04	113.59
Ala58	7.15	124.45	6.94	124.19	7.24	124.79
Val59	7.81	113.77	7.75	114.00	7.75	113.98
Thr60	6.99	108.66	6.87	107.53	7.20	108.74
Asn61	7.47	120.12	7.47	120.11	7.46	120.04
Gln63	8.55	115.06	8.55	115.36	8.55	115.02
Asn64	6.55	112.46	6.54	112.27	6.56	112.57
Thr65	6.88	116.84	6.84	117.14	6.86	117.13
Leu66	9.62	127.89	9.63	127.83	9.60	127.74
Phe67	6.30	115.27	6.27	114.82	6.28	114.89
Ala68	9.12	115.02	9.11	114.90	9.11	114.87
Ile69	7.74	118.30	7.75	118.10	7.75	118.18
Arg71	7.66	114.67	7.60	114.59	7.61	114.51
Leu72	7.72	114.65	7.66	114.91	7.68	114.38
Ile73	7.00	119.20	7.06	119.04	6.98	118.98
Gly74	9.88	115.02	9.84	114.97	9.84	114.97
Arg75	7.94	117.87	7.92	117.98	7.95	117.97
Arg76	8.65	121.09	8.65	121.33	8.60	121.00
Phe77	8.68	122.75	8.67	122.74	8.70	122.69
Gln78	8.16	111.62	8.14	111.50	8.13	111.60
Asp79	7.25	123.38	7.22	123.31	7.21	123.31
Glu80	9.02	127.27	9.03	127.05	9.00	127.38
Glu81	9.13	118.60	9.13	118.51	9.03	118.53
Val82	6.83	119.99	6.80	119.99	6.85	120.04
Gln83	8.09	116.53	8.15	116.60	8.08	116.38
Arg84	7.48	119.50	7.45	119.51	7.46	119.32
Asp85	7.29	121.54	7.26	121.53	7.33	121.65
Val86	8.31	121.92	8.31	121.79	8.30	121.74

Table S3. Chemical shifts of ^{1}H-^{15}N apoNBD$^{1\text{-}392}$ and in complex with GrpE$^{69\text{-}197}$ and GrpE$^{69\text{-}197}$ G122D.

Ser87	7.22	110.70	7.17	110.44	7.21	110.64
Ile88	7.25	116.06	7.23	115.83	7.22	115.81
Met89	7.76	121.97	7.78	121.78	7.74	122.03
Phe91	5.93	115.02	5.88	114.99	5.89	114.93
Lys92	8.93	121.96	9.02	122.41	8.94	121.92
Ile93	7.80	129.03	7.80	129.18	7.81	129.07
Ile94	8.62	120.93	8.62	120.94	8.62	120.93
Ala95	7.42	122.95	7.41	122.94	7.41	122.95
Ala96	8.57	125.95	8.56	125.86	8.56	125.87
Asn98	7.27	114.19	7.30	114.08	7.24	114.12
Gly99	7.77	108.21	7.75	108.17	7.75	108.13
Asp100	7.93	124.25	7.91	124.17	7.91	124.17
Ala101	8.34	121.77	8.35	121.67	8.38	121.71
Trp102	9.54	129.82	9.52	129.77	9.52	129.71
Val103	8.76	115.31	8.81	115.21	8.79	115.28
Glu104	8.45	125.49	8.46	125.82	8.46	125.83
Val105	8.59	126.26	8.56	126.30	8.57	126.78
Lys106	9.22	127.70	9.14	127.56	9.14	127.59
Gly107	8.56	104.65	8.53	104.37	8.53	104.47
Gln108	8.02	121.56	7.98	121.28	7.99	121.35
Lys109	8.54	124.78	8.56	124.41	8.55	124.46
Met110	9.33	123.63	9.32	123.86	9.32	123.77
Ala111	7.51	125.91	7.52	126.14	7.50	126.04
Gln114	7.30	113.62	7.29	113.58	7.28	113.54
Ile115	7.22	119.28	7.22	119.66	7.23	119.32
Ser116	8.32	115.80	8.25	115.60	8.30	115.59
Ala117	8.09	122.23	8.12	121.70	8.10	121.99
Glu118	7.53	115.56	7.52	115.25	7.52	115.34
Val119	6.90	119.34	6.90	119.36	6.91	119.28
Leu120	7.47	118.81	7.49	118.89	7.50	118.83
Lys121	8.84	120.35	8.81	120.31	8.81	120.31
Lys122	7.12	121.04	7.10	120.85	7.10	120.88
Met123	8.08	122.08	8.05	122.03	8.06	122.06
Lys124	8.33	121.06	8.32	120.95	8.31	121.14
Lys125	8.08	119.86	8.09	119.95	8.07	119.80
Thr126	7.93	116.16	7.93	116.42	7.90	116.35
Ala127	7.51	122.74	7.52	122.75	7.51	122.75
Glu128	8.58	120.67	8.61	120.59	8.57	120.56

Table S3. Chemical shifts of ^{1}H-^{15}N apoNBD$^{1-392}$ and in complex with GrpE$^{69-197}$ and GrpE$^{69-197}$ G122D.

Asp129	8.95	121.80	9.09	122.15	8.90	121.70
Tyr130	7.26	120.08	7.25	120.00	7.25	119.95
Leu131	8.61	117.63	8.63	117.42	8.62	117.27
Gly132	8.49	107.72	8.47	107.52	8.48	107.63
Glu133	7.22	116.50	7.17	116.38	7.20	116.36
Val135	9.84	125.59	9.83	125.67	9.84	125.59
Thr136	9.02	113.93	8.97	113.87	8.99	113.84
Glu137	7.75	123.01	7.75	122.92	7.74	122.95
Ala138	8.17	119.18	8.17	118.97	8.17	119.05
Val139	8.77	122.18	8.74	122.03	8.77	122.10
Ile140	7.32	124.57	7.27	124.55	7.29	124.49
Thr141	7.75	116.55	7.76	116.56	7.75	116.55
Val142	7.81	108.72	7.76	108.52	7.78	108.61
Ala144	10.08	124.92	10.00	124.51	10.01	124.71
Tyr145	6.15	106.96	6.11	106.76	6.16	106.96
Phe146	7.24	124.97	7.27	124.96	7.24	124.97
Asn147	9.18	124.95	9.20	124.67	9.13	124.75
Asp148	8.35	117.87	8.30	117.89	8.35	117.85
Ala149	8.09	124.81	8.08	124.81	8.08	124.68
Gln150	8.61	120.11	8.60	120.02	8.59	120.07
Arg151	8.49	121.89	8.44	122.63	8.47	121.88
Gln152	8.79	120.64	8.75	120.50	8.76	120.58
Ala153	8.08	121.14	8.11	120.99	8.10	121.31
Lys155	8.02	123.45	8.01	123.54	8.02	123.45
Asp156	8.69	121.61	8.71	121.68	8.68	121.41
Ala157	7.68	122.30	7.64	122.10	7.66	122.17
Gly158	7.53	103.86	7.49	103.70	7.52	103.80
Arg159	8.07	124.41	8.06	124.46	8.05	124.27
Ile160	8.43	123.93	8.41	123.84	8.40	123.92
Ala161	7.39	120.57	7.44	120.59	7.38	120.56
Gly162	7.78	106.36	7.78	106.31	7.77	106.31
Leu163	8.12	119.83	8.17	119.70	8.13	119.80
Glu164	8.82	124.15	8.80	124.04	8.81	124.07
Val165	8.36	129.18	8.38	129.39	8.36	129.18
Lys166	8.84	130.08	8.78	130.03	8.81	130.02
Arg167	7.08	113.87	7.08	114.03	7.11	114.01
Ile168	8.18	124.82	8.20	124.89	8.18	124.82
Ile169	7.58	119.92	7.60	119.98	7.57	119.72

Table S3. Chemical shifts of ^{1}H-^{15}N apoNBD$^{1-392}$ and in complex with GrpE$^{69-197}$ and GrpE$^{69-197}$ G122D.

Asn170	8.38	119.77	8.32	119.99	8.33	119.55
Glu171	9.05	123.39	9.17	123.80	9.04	123.61
Thr173	6.56	115.68	6.74	116.18	6.53	115.86
Ala174	7.81	123.73	7.83	123.51	7.80	123.59
Ala175	8.16	119.11	8.23	119.20	8.15	119.04
Ala176	7.61	119.37	7.62	119.44	7.57	119.48
Leu177	8.01	118.68	8.06	118.13	8.01	118.71
Leu181	7.86	117.83	7.87	117.58	7.87	117.70
Asp182	9.72	115.51	9.73	115.36	9.72	115.32
Lys183	7.17	119.06	7.19	119.17	7.13	118.95
Gly184	7.83	109.40	7.84	109.64	7.83	109.40
Thr185	8.04	113.73	8.03	113.76	8.04	113.73
Gly186	8.39	112.60	8.39	112.81	8.38	112.50
Asn187	8.13	120.21	8.20	120.50	8.14	120.17
Arg188	8.43	123.08	8.40	123.36	8.49	123.13
Thr189	9.70	121.61	9.74	121.80	9.72	121.79
Ile190	9.40	124.06	9.40	124.41	9.38	124.20
Ala191	8.32	125.02	8.41	124.62	8.34	125.08
Val192	9.09	122.82	9.09	123.08	9.04	122.57
Tyr193	8.58	100.92	8.57	101.25	8.57	100.93
Asp194	8.29	129.72	8.27	129.77	8.31	129.70
Leu195	8.24	126.29	8.25	126.09	8.24	126.29
Gly196	8.65	113.71	8.69	114.10	8.55	113.60
Gly197	9.12	111.08	9.01	110.50	9.09	110.78
Ala199	7.40	121.38	7.36	122.00	7.29	121.19
Phe200	7.74	120.75	7.77	120.96	7.80	120.85
Asp201	8.75	130.35	8.70	129.84	8.73	130.17
Ile202	7.96	118.86	7.96	117.96	7.94	118.72
Ser203	7.96	120.24	7.94	120.15	7.93	120.00
Ile204	8.78	125.77	8.74	125.34	8.76	125.60
Ile205	9.17	126.54	9.13	126.54	9.17	126.23
Glu206	9.26	126.80	9.21	126.07	9.26	126.57
Ile207	9.23	130.08	9.25	129.80	9.21	129.79
Asp208	8.11	127.77	8.10	127.88	8.10	127.47
Glu209	8.58	121.42	8.60	121.63	8.61	121.49
Val210	8.61	126.77	8.64	127.08	8.61	126.88
Asp211	9.21	129.26	9.21	129.16	9.19	129.09
Gly212	8.43	104.09	8.43	103.93	8.43	104.09

Table S3. Chemical shifts of $^{1}H-^{15}N$ apoNBD$^{1-392}$ and in complex with GrpE$^{69-197}$ and GrpE$^{69-197}$ G122D.

Glu213	7.70	121.53	7.68	121.56	7.68	121.41
Lys214	8.50	123.47	8.49	123.71	8.46	123.41
Thr215	8.64	117.05	8.60	117.42	8.63	116.99
Phe216	8.34	120.87	8.32	120.60	8.34	120.87
Glu217	8.85	124.92	8.82	125.52	8.86	124.88
Val218	8.86	126.18	8.98	126.02	8.86	125.96
Leu219	9.13	129.47	9.14	129.06	9.11	129.18
Ala220	7.48	116.73	7.49	116.76	7.46	116.63
Thr221	8.46	111.54	8.44	110.99	8.47	111.47
Asn222	9.11	123.55	9.23	124.16	9.10	123.63
Gly223	10.18	112.91	10.24	113.06	10.18	112.70
Asp224	8.62	119.40	8.62	119.39	8.61	119.57
Thr225	8.70	120.09	8.56	119.33	8.68	119.43
His226	8.91	119.85	8.73	118.49	8.87	119.47
Leu227	6.76	122.86	6.91	124.16	6.74	123.43
Gly228	8.68	109.51	8.41	109.85	8.59	109.63
Gly229	9.01	108.81	8.85	108.44	9.06	108.57
Glu230	9.02	120.00	8.93	118.07	9.05	120.25
Asp231	8.09	116.79	8.16	117.00	8.14	116.78
Ser234	8.29	114.85	8.13	114.44	8.22	114.64
Arg235	7.16	117.98	7.11	118.99	7.18	117.91
Leu236	7.08	120.37	7.04	120.33	7.09	120.30
Ile237	8.74	121.01	8.75	121.32	8.66	120.94
Asn238	8.20	116.11	8.21	116.15	8.22	115.89
Tyr239	7.29	120.80	7.33	120.20	7.35	120.66
Leu240	8.11	119.54	8.04	118.87	8.11	119.54
Val241	8.44	118.21	8.53	119.24	8.51	118.36
Glu242	8.28	122.16	8.30	122.04	8.27	122.01
Glu243	8.57	121.32	8.55	121.27	8.53	121.45
Phe244	8.25	121.71	8.25	121.98	8.26	121.78
Lys245	8.07	120.40	8.06	120.24	8.04	120.28
Lys246	7.79	120.60	7.80	120.94	7.79	120.52
Asp247	7.67	117.10	7.74	117.18	7.67	117.10
Gln248	8.13	113.33	8.12	113.66	8.11	113.35
Gly249	7.71	108.52	7.65	108.20	7.68	108.40
Ile250	6.15	118.59	6.14	118.61	6.14	118.57
Asp251	8.84	127.87	8.80	127.74	8.82	127.76
Leu252	8.99	127.84	9.02	127.87	8.97	127.71

Table S3. Chemical shifts of ^1H-^{15}N apoNBD$^{1-392}$ and in complex with GrpE$^{69-197}$ and GrpE$^{69-197}$ G122D.

Arg253	8.02	115.24	7.99	114.80	8.00	115.03
Asn254	7.16	114.07	7.12	114.06	7.16	113.98
Asp255	7.85	121.96	7.81	121.54	7.84	122.16
Leu257	7.62	119.56	7.68	119.48	7.59	119.80
Ala258	7.51	125.09	7.62	124.15	7.92	125.75
Met259	8.19	114.71	8.10	114.52	8.20	114.44
Gln260	7.73	121.90	7.69	121.85	7.69	121.74
Leu262	8.63	122.41	8.65	121.95	8.66	122.56
Lys263	8.08	121.06	8.07	121.00	8.08	121.01
Glu264	7.15	118.63	7.25	118.36	7.20	119.46
Ala266	8.92	122.89	8.98	123.39	8.38	122.74
Glu267	7.67	120.30	7.70	120.28	7.68	120.06
Lys268	7.37	118.37	7.34	118.50	7.37	118.52
Ala269	8.32	121.85	8.19	121.72	8.34	121.63
Lys270	7.85	116.82	8.05	117.77	8.14	117.64
Ile271	7.39	120.42	7.36	120.61	7.39	120.33
Ser275	7.40	113.31	7.38	113.06	7.39	113.21
Ala276	8.18	125.87	8.12	125.73	8.21	125.87
Gln278	7.45	114.93	7.41	114.67	7.43	114.85
Thr279	8.47	116.16	8.34	116.18	8.40	116.12
Asp280	7.92	123.96	7.94	123.22	7.94	124.01
Val281	9.22	126.88	9.14	127.40	9.18	126.85
Asn282	8.29	127.77	8.32	127.73	8.28	127.61
Leu283	8.78	124.79	8.79	125.20	8.77	124.69
Tyr285	11.93	126.63	12.26	125.98	11.93	126.63
Ile286	7.94	120.88	7.86	120.90	7.94	120.95
Thr287	7.32	112.65	7.29	112.31	7.32	112.39
Ala288	8.33	124.33	8.33	126.11	8.29	124.14
Asp289	7.99	122.53	7.96	122.38	7.96	122.43
Thr291	8.48	109.21	8.50	109.26	8.45	109.10
Gly292	7.73	111.57	7.71	111.15	7.70	111.47
Lys294	8.08	118.82	8.10	118.02	8.06	118.76
His295	8.41	119.99	8.66	120.04	8.41	119.98
Met296	8.06	123.43	7.92	122.81	8.02	123.11
Asn297	9.25	129.59	9.22	129.76	9.22	129.58
Lys299	8.50	129.95	8.41	129.38	8.49	129.80
Val300	8.98	127.15	8.99	127.02	8.98	126.77
Thr301	7.74	116.07	7.69	115.95	7.75	116.14

Table S3. Chemical shifts of ^{1}H-^{15}N apoNBD$^{1-392}$ and in complex with GrpE$^{69-197}$ and GrpE$^{69-197}$ G122D.

Arg302	8.68	123.82	8.66	123.70	8.67	123.72
Ala303	8.37	120.34	8.28	120.56	8.34	120.16
Lys304	7.94	120.61	7.96	120.91	7.96	120.71
Leu305	7.89	121.31	7.86	121.28	7.85	121.32
Glu306	8.67	116.69	8.66	116.99	8.63	116.64
Ser307	7.61	113.50	7.69	113.90	7.60	113.56
Val309	7.28	106.11	7.19	106.15	7.23	105.97
Glu310	7.61	126.43	7.36	128.52	7.57	126.43
Asp311	8.72	115.11	8.72	115.42	8.72	115.08
Leu312	7.05	121.73	6.98	121.54	7.00	121.59
Val313	7.34	117.47	7.37	117.32	7.34	117.25
Asn314	8.47	121.23	8.42	121.93	8.49	121.51
Arg315	7.81	122.18	7.82	122.71	7.80	122.47
Ser316	7.34	115.49	7.33	115.17	7.33	115.20
Ile317	6.97	119.40	7.04	119.45	6.93	118.96
Glu318	7.33	119.28	7.37	119.99	7.26	119.27
Leu320	6.86	116.41	6.98	116.18	6.90	116.24
Lys321	7.46	117.45	7.38	117.99	7.38	117.93
Val322	7.29	120.07	7.29	120.07	7.31	120.01
Ala323	8.01	121.98	8.03	122.14	7.95	122.11
Leu324	7.65	115.46	7.65	115.44	7.62	115.33
Gln325	7.62	119.27	7.58	119.43	7.61	119.43
Ala327	7.82	120.17	7.82	119.98	7.85	119.81
Gly328	7.84	109.02	7.81	108.97	7.79	108.97
Leu329	7.90	119.27	7.87	119.22	7.84	119.01
Ser330	9.15	116.61	9.13	116.44	9.13	116.40
Val331	8.60	119.18	8.59	119.00	8.60	118.99
Ser332	7.58	113.62	7.56	113.51	7.57	113.60
Asp333	7.67	120.11	7.65	120.04	7.65	120.01
Ile334	7.03	118.91	7.09	119.00	7.03	118.91
Asp335	8.93	128.21	8.91	127.78	8.91	127.84
Asp336	7.13	114.97	7.14	114.85	7.11	114.82
Val337	8.70	121.69	8.80	121.66	8.72	121.61
Ile338	8.61	121.14	8.62	121.27	8.57	121.04
Leu339	6.88	122.19	6.83	122.05	6.87	122.02
Val340	9.23	124.96	9.29	124.67	9.25	124.82
Gly341	7.33	109.92	7.41	109.92	7.43	109.99
Gly342	8.88	113.68	8.94	113.87	8.89	114.50

Table S3. Chemical shifts of ^{1}H-^{15}N apoNBD$^{1-392}$ and in complex with GrpE$^{69-197}$ and GrpE$^{69-197}$ G122D.

Thr344	7.87	108.01	7.88	107.79	7.90	107.93
Arg345	7.01	117.01	7.01	117.26	7.01	117.09
Met348	9.13	117.04	9.17	116.86	9.16	117.03
Val349	6.96	118.17	6.95	118.09	6.92	117.96
Gln350	7.15	116.75	7.21	116.61	7.14	116.63
Lys351	8.35	118.77	8.44	118.79	8.35	118.58
Lys352	7.83	120.21	7.91	119.81	7.85	120.36
Val353	8.00	120.31	7.99	120.17	7.95	120.32
Ala354	8.12	123.02	8.15	123.14	8.14	123.11
Glu355	8.38	119.15	8.45	119.21	8.40	118.99
Phe356	7.76	120.36	7.80	120.30	7.76	120.36
Phe357	8.07	112.05	8.05	111.75	8.06	111.99
Gly358	8.19	109.60	8.20	109.65	8.19	109.60
Lys359	7.39	116.31	7.35	116.46	7.36	116.25
Arg362	8.83	121.53	8.89	121.66	8.85	121.65
Lys363	8.39	117.74	8.39	117.29	8.40	117.46
Asp364	9.08	117.79	9.05	117.91	9.03	117.84
Val365	6.94	120.00	6.93	118.10	6.95	119.76
Asn366	8.40	126.61	8.35	126.61	8.36	126.62
Ile373	8.06	116.83	8.07	116.50	8.08	116.71
Gly374	8.25	107.38	8.29	107.46	8.24	107.28
Ala375	7.98	123.93	7.94	123.63	7.98	123.93
Val377	8.49	122.64	8.54	122.87	8.49	122.64
Gln378	8.10	119.77	8.08	119.67	8.09	119.70
Gly379	8.26	107.91	8.20	107.80	8.25	107.88
Gly380	8.10	108.73	8.12	109.18	8.10	108.84
Val381	7.55	119.21	7.51	119.45	7.56	118.85
Leu382	7.42	120.73	7.49	120.59	7.42	120.73
Thr383	7.65	112.89	7.61	113.02	7.62	112.83
Gly384	7.96	110.95	7.94	110.90	7.93	110.83
Asp385	8.11	121.50	8.12	121.37	8.11	121.50
Val386	7.84	120.63	7.85	120.57	7.83	120.56
Lys387	8.27	125.54	8.21	125.22	8.24	125.32
Leu390	8.10	126.27	8.12	126.02	8.11	126.00
Leu391	8.04	124.78	7.99	124.45	8.01	124.56
Leu392	7.62	129.69	7.54	129.24	7.59	129.45

Table S4. Chemical shifts of ^{1}H-^{15}N apoNBD$^{1-392}$ and in complex with GrpE$^{69-197}$ and GrpE$^{69-197}$ G122D.

Table S4. Chemical shifts of ^{1}H-^{15}N apoNBD$^{1-392}$ and in complex with GrpE$^{69-197}$ and GrpE$^{69-197}$ G122D.

Residue	ADP-NBD$^{1-392}$		ADP-NBD$^{1-392}$/GrpE$^{69-197}$	
	^{1}H (ppm)	^{15}N (ppm)	^{1}H (ppm)	^{15}N (ppm)
Lys3	8.94	122.04	8.95	122.07
Ile4	7.96	122.39	7.97	122.33
Ile5	7.45	120.16	7.45	120.16
Gly6	8.94	109.53	8.98	109.55
Ile7	8.51	123.39	8.53	123.36
Asp8	8.03	124.24	-	-
Leu9	6.45	129.59	-	-
Cys15	8.49	124.05	8.47	124.14
Val16	9.16	128.38	-	-
Ala17	8.96	128.15	8.94	128.07
Ile18	8.39	113.45	8.42	113.35
Met19	8.20	121.03	8.22	121.01
Asp20	8.29	128.19	8.31	128.25
Gly21	8.13	114.24	8.13	114.26
Thr22	8.36	117.35	8.39	117.43
Thr23	7.58	118.98	7.60	119.07
Arg25	9.05	124.61	9.07	124.64
Val26	8.33	127.76	8.33	127.77
Leu27	8.02	126.21	8.03	126.54
Glu28	8.03	120.18	8.05	120.12
Asn29	7.95	118.33	7.98	118.10
Ala30	8.29	121.68	8.33	121.72
Glu31	7.64	116.50	7.60	116.37
Gly32	8.06	108.26	8.08	108.32
Asp33	7.75	119.63	7.77	119.71
Arg34	9.10	123.36	9.08	123.25
Thr35	7.29	108.27	7.32	110.05
Thr36	9.18	117.68	9.13	117.73
Ser38	9.42	125.20	9.41	124.29
Ile39	8.29	126.57	8.37	127.07
Ile40	8.41	127.78	8.55	128.51
Ala41	9.23	126.94	9.20	126.79
Tyr42	7.76	120.88	7.76	120.92
Thr43	8.53	114.54	8.57	114.86
Asp45	7.80	116.27	7.83	116.29
Gly46	7.62	107.44	7.61	107.30

Table S4. Chemical shifts of ^{1}H-^{15}N apoNBD$^{1-392}$ and in complex with GrpE$^{69-197}$ and GrpE$^{69-197}$ G122D.

Glu47	7.47	120.87	7.46	120.78
Thr48	8.46	120.25	8.47	120.45
Leu49	9.10	127.34	9.08	127.24
Val50	8.41	120.64	8.42	120.43
Gly51	8.52	113.89	8.62	113.91
Gln52	10.40	129.40	10.46	129.07
Ala54	6.39	116.26	6.45	116.26
Lys55	8.30	123.66	8.35	123.38
Arg56	7.88	114.44	7.97	115.41
Gln57	7.06	112.07	6.88	112.61
Ala58	7.09	124.07	7.15	124.28
Thr60	6.96	107.34	6.98	108.59
Asn61	7.39	119.95	-	-
Gln63	8.58	115.83	8.62	116.79
Asn64	6.46	112.25	6.53	112.41
Thr65	6.85	116.28	6.96	116.12
Leu66	9.57	127.99	9.60	127.92
Phe67	6.33	116.65	6.40	116.61
Ala68	9.30	115.80	9.12	117.08
Ile69	7.72	118.52	7.74	118.39
Arg71	7.68	114.42	7.67	114.39
Leu72	7.66	114.28	7.71	114.41
Ile73	6.92	119.11	6.93	118.93
Gly74	9.84	114.68	9.86	114.94
Arg75	7.98	118.11	7.95	117.94
Arg76	8.73	121.09	8.72	121.09
Phe77	8.71	122.76	8.71	122.79
Gln78	8.10	111.68	8.13	111.66
Asp79	7.20	123.35	7.22	123.37
Glu80	9.03	127.92	9.03	127.88
Glu81	9.01	119.27	9.02	118.75
Val82	6.78	119.35	6.90	118.75
Gln83	8.04	116.21	8.07	116.41
Arg84	7.45	119.42	7.45	119.40
Asp85	7.34	120.97	7.33	121.12
Val86	8.25	122.02	8.26	121.78
Ser87	7.19	110.60	7.24	110.67
Ile88	7.20	116.45	7.18	116.42
Met89	7.68	121.89	7.67	122.14
Phe91	5.93	114.90	5.91	115.02

Table S4. Chemical shifts of ^{1}H-^{15}N apoNBD$^{1-392}$ and in complex with GrpE$^{69-197}$ and GrpE$^{69-197}$ G122D.

Lys92	8.80	121.77	8.78	121.66
Ile93	7.75	128.80	7.79	128.93
Ile94	8.60	120.86	8.60	120.97
Ala95	7.43	122.94	7.43	122.97
Ala96	8.52	125.91	8.56	126.01
Asn98	7.24	114.21	7.25	114.18
Gly99	7.74	108.19	7.76	108.20
Asp100	7.91	124.23	7.92	124.26
Ala101	8.32	121.72	8.36	121.61
Trp102	9.52	129.71	9.53	129.82
Val103	8.70	114.06	8.78	114.65
Glu104	8.34	124.72	8.34	124.59
Val105	8.50	125.78	8.58	126.28
Lys106	9.37	128.08	9.30	127.33
Gly107	8.54	104.69	8.55	104.62
Gln108	7.99	121.61	8.00	121.51
Lys109	8.51	124.95	8.54	124.79
Met110	9.29	123.46	9.32	123.65
Ala111	7.48	125.91	7.50	125.97
Gln114	7.29	113.59	7.29	113.60
Ile115	7.20	119.16	7.16	119.06
Ala117	8.06	122.01	8.08	122.08
Glu118	7.44	115.48	7.51	115.20
Val119	6.83	119.43	6.88	119.38
Leu120	7.54	119.12	7.54	119.03
Lys121	8.77	120.31	8.80	120.18
Lys122	7.03	121.03	7.01	121.17
Met123	7.99	121.73	7.99	121.63
Lys124	8.28	121.02	8.31	121.13
Lys125	7.97	119.85	7.96	120.07
Thr126	7.89	116.19	7.92	116.13
Ala127	7.48	122.72	7.48	122.79
Glu128	8.55	120.60	8.56	120.64
Asp129	8.93	121.80	8.92	121.83
Tyr130	7.22	120.06	7.24	120.09
Leu131	8.57	117.61	8.60	117.61
Gly132	8.44	107.73	8.47	107.79
Glu133	7.19	116.55	7.20	116.52
Val135	9.82	125.55	9.84	125.58
Thr136	8.99	113.83	9.02	113.86

Table S4. Chemical shifts of ^{1}H-^{15}N apoNBD$^{1-392}$ and in complex with GrpE$^{69-197}$ and GrpE$^{69-197}$ G122D.

Glu137	7.70	123.02	7.73	122.97
Ala138	8.14	119.11	8.16	119.14
Val139	8.77	122.12	8.76	122.10
Ile140	7.29	124.41	7.32	124.55
Thr141	7.75	116.45	7.74	116.48
Val142	7.81	108.48	7.80	108.72
Ala144	10.08	124.84	9.86	124.75
Tyr145	6.16	106.98	6.26	107.28
Phe146	7.23	125.28	7.22	124.98
Asn147	9.11	124.91	9.10	124.72
Asp148	8.32	117.69	8.33	117.78
Ala149	8.05	124.91	8.07	124.88
Gln150	8.61	120.19	8.61	120.10
Arg151	8.47	121.92	8.48	121.90
Gln152	8.74	120.57	8.75	120.58
Ala153	8.08	121.14	8.07	121.14
Lys155	7.98	123.33	8.01	123.46
Asp156	8.67	121.88	8.64	121.66
Ala157	7.67	122.36	7.66	122.29
Gly158	7.49	103.66	7.51	103.83
Arg159	8.06	124.51	8.06	124.41
Ile160	8.37	123.87	8.41	123.94
Ala161	7.34	120.60	7.37	120.62
Gly162	7.75	106.36	7.77	106.34
Leu163	8.11	119.87	8.09	119.81
Glu164	8.80	124.23	8.81	124.14
Val165	8.31	129.06	8.35	129.17
Lys166	8.82	129.89	8.82	129.83
Arg167	7.08	114.32	7.14	114.10
Ile168	8.17	125.13	8.19	124.86
Ile169	7.47	119.82	7.47	120.15
Asn170	8.33	119.49	8.35	119.76
Glu171	8.81	122.20	8.78	122.03
Ala174	7.83	123.79	7.80	123.72
Ala175	8.19	118.86	8.17	118.76
Ala176	7.61	119.61	7.63	119.54
Leu177	7.84	118.35	7.77	118.36
Leu181	7.75	117.91	7.74	118.06
Asp182	9.69	116.26	9.49	117.16
Lys183	7.22	119.05	7.20	119.20

Table S4. Chemical shifts of ^{1}H-^{15}N apoNBD$^{1-392}$ and in complex with GrpE$^{69-197}$ and GrpE$^{69-197}$ G122D.

Gly184	7.75	109.10	7.81	109.50
Thr185	8.01	113.72	8.01	113.72
Gly186	8.42	112.47	8.40	112.42
Asn187	8.11	120.32	8.09	120.17
Arg188	8.48	123.76	8.54	124.43
Thr189	9.59	121.45	9.58	121.82
Ile190	9.38	123.89	9.40	123.89
Ala191	8.28	124.68	8.29	124.70
Val192	9.01	122.79	9.01	122.74
Tyr193	8.43	132.64	-	-
Asp194	8.43	129.58	8.65	128.80
Leu195	7.95	127.16	-	-
Asp201	9.38	130.53	9.20	130.60
Ile202	7.89	119.36	7.93	119.42
Ser203	7.75	120.01	7.78	120.07
Ile204	8.70	125.98	8.75	125.61
Ile205	9.10	127.16	9.06	127.08
Glu206	9.23	127.76	9.21	127.74
Ile207	9.20	130.86	9.24	130.75
Asp208	8.12	127.69	8.14	127.76
Glu209	8.65	121.70	8.63	121.68
Val210	8.58	126.74	8.58	126.47
Asp211	9.19	129.37	9.21	129.39
Gly212	8.42	104.10	8.44	104.12
Glu213	7.67	121.47	7.69	121.51
Lys214	8.51	124.15	8.52	124.26
Thr215	8.59	116.33	8.60	116.90
Phe216	8.33	120.71	8.37	120.77
Glu217	8.84	124.65	8.85	124.74
Val218	8.89	126.89	8.88	126.67
Leu219	9.09	129.71	9.10	129.58
Ala220	7.37	116.37	7.37	116.34
Thr221	8.46	112.63	8.45	112.50
Asn222	9.17	124.47	9.15	124.84
Gly223	10.30	113.00	-	-
Asp224	8.85	120.44	8.83	120.42
Thr225	8.59	118.79	8.58	118.97
His226	8.95	121.01	8.90	121.50
Leu227	6.72	124.72	6.71	123.63
Gly228	8.64	107.56	8.40	107.80

Table S4. Chemical shifts of ^{1}H-^{15}N apoNBD$^{1-392}$ and in complex with GrpE$^{69-197}$ and GrpE$^{69-197}$ G122D.

Gly229	9.98	108.56	9.90	108.60
Glu230	9.72	122.96	-	-
Asp231	7.87	117.86	7.88	117.84
Ser234	7.87	113.51	7.86	113.87
Arg235	7.29	118.72	7.33	117.99
Leu236	7.33	121.26	7.31	121.16
Ile237	9.11	121.49	8.96	122.58
Asn238	7.96	116.33	7.94	116.22
Leu240	8.22	119.83	8.18	119.21
Val241	8.48	118.32	8.50	118.45
Glu242	8.19	122.09	8.20	121.92
Glu243	8.55	121.55	8.56	121.35
Lys245	8.04	120.31	8.04	120.36
Lys246	7.75	120.64	7.77	120.55
Asp247	7.65	117.05	7.68	117.02
Gln248	8.11	113.34	8.12	113.33
Gly249	7.65	108.43	7.67	108.44
Ile250	6.12	118.57	6.13	118.57
Asp251	8.82	127.83	8.83	127.84
Leu252	8.81	127.73	8.80	127.83
Arg253	7.96	114.79	7.99	115.30
Asn254	7.10	114.07	7.08	114.10
Asp255	7.75	121.99	7.83	121.98
Leu257	7.48	117.63	7.55	118.09
Ala258	7.28	123.69	-	-
Met259	8.20	115.23	8.18	114.62
Gln260	7.95	122.79	-	-
Leu262	8.84	122.29	8.91	121.98
Lys263	8.23	121.71	8.25	121.82
Glu264	7.16	119.49	7.12	119.44
Ala265	8.05	121.23	8.06	121.33
Ala266	9.16	122.18	9.05	122.85
Glu267	7.28	118.60	7.27	118.60
Lys268	7.36	117.33	7.37	117.41
Ala269	8.04	122.14	8.06	122.29
Lys270	8.03	118.43	8.00	118.46
Ile271	7.77	120.11	7.81	120.06
Ser275	7.71	113.25	7.71	113.30
Ala276	8.16	125.91	8.17	125.87
Gln277	8.64	116.01	8.65	115.98

Table S4. Chemical shifts of ^{1}H-^{15}N apoNBD$^{1-392}$ and in complex with GrpE$^{69-197}$ and GrpE$^{69-197}$ G122D.

Gln278	7.43	115.01	7.44	114.96
Thr279	8.44	116.37	8.48	116.35
Asp280	7.87	123.42	7.87	123.35
Val281	9.16	126.42	9.18	126.43
Asn282	8.31	127.77	8.29	127.77
Leu283	8.76	124.58	8.77	124.72
Ile286	7.89	120.78	7.90	120.62
Thr287	7.30	112.65	7.30	112.65
Ala288	8.31	124.29	8.32	124.32
Asp289	7.94	122.53	7.97	122.54
Thr291	8.45	109.20	8.47	109.20
Gly292	7.69	111.55	7.71	111.57
Lys294	8.05	118.80	8.07	118.82
His295	8.38	119.91	8.40	119.97
Met296	8.01	123.35	8.04	123.38
Asn297	9.21	129.56	9.24	129.58
Ile298	8.36	125.22	8.37	125.19
Lys299	8.45	129.95	8.47	129.90
Val300	8.98	126.98	8.98	126.82
Thr301	7.68	115.99	7.70	115.95
Arg302	8.68	123.65	8.67	123.75
Ala303	8.34	120.48	8.36	120.44
Lys304	7.90	120.80	7.93	120.87
Leu305	7.82	120.99	7.83	120.96
Glu306	8.68	116.77	8.69	116.71
Ser307	7.58	113.25	7.59	113.34
Val309	7.23	106.22	7.29	106.00
Glu310	7.53	126.45	7.54	126.53
Asp311	8.62	114.78	8.63	114.79
Leu312	7.05	121.89	7.08	121.99
Val313	7.15	117.35	7.17	117.45
Asn314	8.42	120.79	8.39	121.01
Arg315	7.74	122.64	7.79	122.73
Ser316	7.25	114.42	7.33	114.48
Ile317	6.96	119.71	6.96	119.55
Glu318	7.30	119.13	7.28	119.49
euN20L	6.84	116.49	6.85	116.84
Lys321	7.46	117.59	7.40	117.41
Val322	7.33	120.21	7.31	120.14
Ala323	8.02	122.40	8.01	122.44

Table S4. Chemical shifts of ^1H-^{15}N apoNBD$^{1-392}$ and in complex with GrpE$^{69-197}$ and GrpE$^{69-197}$ G122D.

Leu324	7.55	115.43	7.51	115.46
Gln325	7.59	119.19	7.60	119.31
Ala327	7.79	120.16	7.80	120.33
Gly328	7.81	109.09	7.82	108.97
Leu329	7.86	119.28	7.89	119.27
Ser330	9.10	116.47	9.08	116.42
Val331	8.61	119.58	8.64	119.64
Ser332	7.58	113.72	7.62	113.95
Asp333	7.64	120.05	7.67	120.02
Ile334	7.04	119.15	7.00	119.15
Asp335	8.89	128.08	8.92	128.04
Asp336	7.09	114.94	7.10	114.97
Val337	8.68	121.77	8.72	121.78
Val340	9.20	125.33	9.14	125.18
Gly341	6.99	110.12	6.98	111.18
Gly342	9.50	115.73	9.70	115.60
Gln343	6.62	115.86	6.56	115.72
Thr344	7.47	109.24	7.41	109.35
Arg345	6.62	117.24	6.64	117.25
Met346	6.79	118.74	6.85	118.72
Met348	9.25	117.35	9.17	117.06
Val349	6.96	117.63	6.93	117.95
Gln350	6.99	116.46	7.03	116.40
Lys351	8.27	118.88	8.28	118.87
Lys352	7.91	119.97	7.89	119.95
Ala354	8.00	122.86	8.02	122.88
Glu355	8.30	118.98	8.32	118.84
Phe357	8.04	112.03	8.05	112.04
Gly358	8.11	109.67	8.17	109.72
Lys359	7.36	116.22	7.36	116.21
Arg362	8.85	121.79	8.89	121.66
Lys363	8.46	117.98	8.45	117.92
Asp364	9.08	117.70	9.05	117.51
Val365	6.73	118.03	6.90	117.60
Ile373	7.90	116.62	8.02	116.75
Gly374	7.87	106.34	-	-
Ala375	7.78	123.42	7.80	123.25
Ala376	8.07	122.27	8.10	122.40
Val377	8.49	122.48	8.47	122.57
Gln378	7.98	119.57	8.03	119.53

Table S4. Chemical shifts of ^{1}H-^{15}N apoNBD$^{1-392}$ and in complex with GrpE$^{69-197}$ and GrpE$^{69-197}$ G122D.

Gly379	8.25	108.17	8.26	107.95
Gly380	8.04	108.72	8.08	108.83
Val381	7.48	118.74	7.46	118.88
Leu382	7.44	120.83	7.43	120.66
Thr383	7.67	111.78	7.66	112.21
Gly384	7.81	110.71	7.88	110.79
Asp385	8.04	121.02	8.03	121.11
Val386	7.53	120.63	7.50	120.78
Lys387	8.30	125.68	8.29	125.57
Asp388	8.07	120.57	8.07	120.73
Val389	7.73	120.31	7.77	120.44
Leu390	7.96	126.78	-	-
Leu391	8.02	124.52	8.03	124.44
Leu392	7.82	130.19	7.78	130.11
